# Single-molecule tracking of DNMT1 in living cells reveals its cell cycle dynamics and its redistribution upon drug treatment

**DOI:** 10.1093/nar/gkag089

**Published:** 2026-02-05

**Authors:** Eliza S Lee, Ella R Tommer, Paul B Rothman, Sarah V Middleton, Daniel T Youmans, Thomas R Cech

**Affiliations:** BioFrontiers Institute, University of Colorado Boulder, Boulder, CO 80303, USA; Department of Biochemistry, University of Colorado Boulder, Boulder, CO 80303,USA; Howard Hughes Medical Institute, University of Colorado Boulder, Boulder, CO 80303, USA; BioFrontiers Institute, University of Colorado Boulder, Boulder, CO 80303, USA; Department of Biochemistry, University of Colorado Boulder, Boulder, CO 80303,USA; BioFrontiers Institute, University of Colorado Boulder, Boulder, CO 80303, USA; Department of Biochemistry, University of Colorado Boulder, Boulder, CO 80303,USA; John Hopkins School of Medicine, Baltimore, MD 21205, USA; Department of Biochemistry, University of Colorado Boulder, Boulder, CO 80303,USA; Department of Molecular, Cellular and Developmental Biology, University of Colorado Boulder, Boulder, CO 80303, USA; BioFrontiers Institute, University of Colorado Boulder, Boulder, CO 80303, USA; Department of Biochemistry, University of Colorado Boulder, Boulder, CO 80303,USA; John Hopkins School of Medicine, Baltimore, MD 21205, USA; BioFrontiers Institute, University of Colorado Boulder, Boulder, CO 80303, USA; Department of Biochemistry, University of Colorado Boulder, Boulder, CO 80303,USA; Howard Hughes Medical Institute, University of Colorado Boulder, Boulder, CO 80303, USA

## Abstract

DNMT1 is a methyltransferase that restores 5-methylcytidine marks on newly replicated DNA and is required for maintaining epigenetic inheritance. Using Halo-tagged DNMT1 and highly inclined thin illumination (HiLo) microscopy, we show that DNMT1 mobility in living human cells changes under a variety of conditions. DNMT1 molecules become increasingly bound to chromatin in the S phase of the cell cycle, but surprisingly only ∼ 12% chromatin-bound DNMT1 is sufficient to maintain DNA methylation. Upon treatment with small molecule inhibitors, GSK-3484862 (GSK), 5-azacytidine (5-azaC) and decitabine (5-aza-deoxyC), *in vivo* DNMT1 dynamics are greatly altered. Unexpectedly, treatment of cells with GSK, a non-covalent inhibitor, causes binding of DNMT1 to chromatin similar to that observed upon treatment with 5-azaC and decitabine, covalent inhibitors. 5-azaC inhibition of DNMT1 dynamics occurs during the S phase of the cell cycle. Unexpectedly, mutations in the disordered, Asp- and Glu-rich *N*-terminal region of DNMT1 dramatically decrease its mobility and increase chromatin binding. Collectively, our work using live cell single molecule imaging quantifies the molecular dynamics of DNMT1 and how this relates to its function under physiological conditions and upon drug treatment. Understanding the dynamics of DNMT1 *in vivo* provides a framework for developing better therapeutics that target DNMT1.

## Graphical Abstract

**Graphical Abstract:**
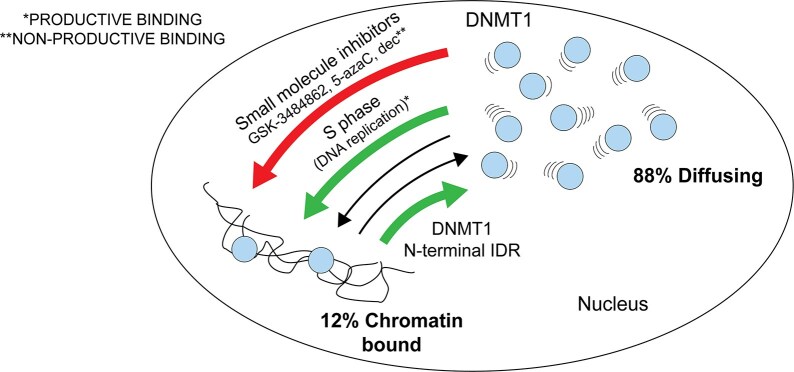

## Introduction

The epigenetic status of a cell is established and maintained by DNA methylation and histone modifications. DNA methyltransferase 1 (DNMT1) methylates CpG sequences of hemi-methylated DNA, maintaining epigenetic inheritance. Typically, DNMT1 methylates CpG islands in promoter regions to suppress the expression of the downstream gene [1]. DNA methylation is also critical for suppressing retrotransposon element expression [2–5]. DNA methylation is crucial for many developmental processes; in particular, for early embryogenesis [3, 6, 7] and gametogenesis [8, 9]. Importantly, dysregulation of DNMT1 frequently leads to diseases, and abnormal methylation is a hallmark of many cancers [10–12].

Several studies have begun to explore the subcellular localization of DNMT1 and how this relates to its function. Similar to many other chromatin-modifying factors, the subnuclear localization of DNMT1 changes throughout the cell cycle. During the G1 phase of the cell cycle, DNMT1 is nucleoplasmic, whereas during mitosis, DNMT1 associates with the dividing chromosomes [13]. During the G2 phase, there is some evidence to suggest that DNMT1 associates with chromatin factors [13]. In S phase, DNMT1 interacts with replication forks and methylates the newly synthesized DNA [13], and loss of this interaction greatly impacts DNA methylation. DNMT1 can associate with S phase factors in multiple but redundant ways, including interactions with the sliding clamp of the DNA replication machinery PCNA [14–16] and binding through its partner protein URHF1 [17, 18]. Although the dynamics of DNMT1 during the cell cycle have been explored by bulk cell imaging, quantifying the number of DNMT1 molecules that redistribute during the cell cycle requires single molecule tracking.

While the C-terminus of DNMT1 confers the methyltransferase enzymatic activity, its *N*-terminal domains are regulatory. In particular, the Replication Foci Targeting Sequence (RFTS) is required for recruitment to the replication fork during S phase [14] and is thought to be required for the processivity of the DNMT1 methyltransferase enzyme [19]. Notably, the *N*-terminal domain contains an intrinsically disordered region (IDR) from residues 100 to 400. Additionally, a recent study identified an alpha-helical domain from residues 22 to 91 of DNMT1 that precedes the disordered region, which is required for binding to the transcriptional repressor DMAP1 protein [20–22]. Interestingly, independent of DNA methylation activity, the *N*-terminus of DNMT1 is required for its function as a transcription factor, possibly through DMAP1 [21, 23]. In aggregate, evidence suggests that the *N*-terminal region of DNMT1 regulates its roles as both methyltransferase and transcriptional repressor, so their effect on the mobility of DNMT1 within the cell is of interest.

Various small molecule inhibitors of DNMT1 have been developed as therapeutics for cancers including acute myeloid leukemia (AML) and myelodysplastic syndromes [24–28]. One such inhibitor studied here is the cytosine analog, 5-azaC. 5-azaC is converted in cells to the deoxynucleotide, which is incorporated into the target DNA and acts as a suicide inhibitor of DNMT1 during DNA methylation [27, 29–31]. The deoxy analog of 5-azaC, decitabine, is also approved for cancer therapy [24, 26–28]. Another inhibitor, GSK-3484862 (GSK), reduces DNA methylation by targeting DNMT1 for degradation through the proteasomal pathway [32, 33]. The crystal structures of DNMT1 with the related GSK inhibitors GSK-3735967 and GSK-3685032 (containing the same functional groups but differing in inhibitor activity) suggest that these drugs act on DNMT1 by intercalating between the bases of its DNA substrate and consequently wedge the DNA substrate apart at the active site [34, 35]. Unlike earlier generations of DNMT1 inhibitors, GSK compounds are non-nucleosidyl inhibitors and specifically inhibit DNMT1 as they do not directly target the related *de novo* DNA methyltransferases DNMT3A and DNMT3B [32]. How these small molecule inhibitors influence the dynamics of DNMT1 *in vivo* remains an open question.

Currently, most DNMT1 studies draw conclusions from bulk or population effects. They do not focus on DNMT1 at a single molecule level. Similarly, small molecule inhibitor studies have mostly focused on the effects of long-term treatment (>24 h) [32–36]. The acute effects of these inhibitors on DNMT1 dynamics (and also function) at early timepoints have not been studied. Early timepoints can reveal the direct mechanism of drug action, whereas later timepoints reflect both direct and indirect cellular responses.

To address these challenges, we applied a CRISPR genome-editing and single molecule live cell imaging approach previously developed for investigating the mobility of telomerase and chromatin-modifying factors [37–39] to study DNMT1 dynamics. Using cell lines expressing Halo-tagged DNMT1 under the endogenous promoter, we found that DNMT1 mobility changes during cell cycle progression. Importantly, during S phase, a subset of DNMT1 molecules become more chromatin-bound, consistent with DNMT1 association with the DNA replication machinery. In contrast, DNMT1 becomes more mobile in G2 phase. Moreover, we show that acute treatment with three small molecule inhibitors dramatically decreases DNMT1 mobility and increases its chromatin binding, which precedes DNMT1 protein degradation. Collectively, these results connect the dynamics of DNMT1 at single-molecule resolution with both its biological function and its pharmacology.

## Materials and methods

### Cell culture and small molecule inhibition of DNMT1

All cells were maintained in DMEM media supplemented with 10% fetal bovine serum, 1X penicillin-streptomycin and 1X GlutaMAX-I at 37°C with 5% CO_2_. Note that the parental U2OS cell line (generous gift from the Rippe lab, University of Heidelberg, Germany) contains a lacO-array incorporated into the genome [40] that has enriched K27 methylation [41], but this is not pertinent to the current study.

To express DNMT1 *N*-terminal mutants, 100 ng of plasmid was transfected into U2OS cells plated onto 35 mm imaging dishes (Ibdi, USA, #81158) using GenJet In Vitro DNA Transfection Reagent for U2OS Cells (GenJet, USA, #SL100489-OS), according to manufacturer’s instructions. 18-24 h post-transfection, cells were washed and prepared for live cell single molecule imaging as described below.

For small molecule inhibition experiments, U2OS cells were treated with 4 µM GSK (MedChemExpress, USA, #HY-135146), 5-azaC (Sigma-Aldrich, #A2385) or decitabine (Selleck Chemicals, USA, #S1200) (all dissolved in DMSO) or with DMSO only for the time indicated and harvested. For fixed cell imaging of DNMT1, cells plated on coverslips were treated with the drug of interest for the time indicated. 500 nM Halo ligand JF549 dye (Janelia Research Campus, Howard Hughes Medical Institute, Ashburn, USA) was added to coverslips and incubated for 5 min, washed twice with PBS and fixed with 4% formaldehyde (Electron Microscopy Sciences, USA, #15686) for 15 min. Next, coverslips were washed with PBS and mounted with DAPI fluoromount-G solution (Southern Biotech, USA, #0100-20). The coverslips were imaged on the DeltaVision epifluorescence widefield scope.

### Genome editing of U2OS cells

To generate DNMT1-Halo cell lines, DNMT1 cDNA exons 4-39 linked to a Halo tag were knocked into the endogenous *DNMT1* locus at exon 4. CRISPR editing of DNMT1-Halo clones was adapted from the pX330 cloning protocol available from the Genetically Engineered Models Center at the Whitehead Institute. U2OS cells at ∼80% confluency in 6-well plates were transfected with 1 µg of each vector (pX330 and DNMT1-Halo targeting vector) using jetPRIME transfection reagent (Polyplus-Satorius, USA, #101000015) per manufacturer’s instructions. Transfection controls included GFP (negative control), targeting vector alone (control for extrachromosomal maintenance), and a DNMT1-Halo overexpression vector under a CMV promoter (positive control for Halo signal). Transfection reagents were removed after 18 h, and cells were expanded to a T-25 flask. Edited cells were selected with 10 µg/ml puromycin in complete DMEM for 7 days (cells were given fresh selection media daily). After initial selection, cells were stained with 500 nM JF549 Halo ligand for 5 min, washed 3X with complete DMEM media and sorted using a BD FACS Aria flow cytometer. JF549-positive cells were sorted into a gross edited population. To obtain monoclonal edited cell lines, the gross edited population was stained with JF549 Halo ligand and sorted one cell at a time into 96-well plates. Following growth and expansion, monoclonal edited populations were screened for DNMT1-Halo knock-in using microscopy, genomic DNA PCR, and western blotting.

### Immunoblotting and fluorophore staining of gels

For all immunoblots, 60 mm dishes of U2OS cells were washed twice with 1X PBS, harvested with 1X Laemmli buffer and the sample was incubated at 65°C for 5 min. The sample was separated on an SDS-PAGE gel, transferred onto a nitrocellulose membrane and blotted with specific antibodies diluted to 1:1000: mouse monoclonal FLAG-M2 (Sigma, #F3165), DNMT1 (Abcam, #ab13537), PARP1 (Santa Cruz, F-2, # sc-8007) and actin (Sigma Aldrich, AC-15, #A5441).

To visualize the DNMT1-Halo-FLAG protein via fluorescence, we added 500 nM of JF646 Halo ligand dye in DMEM and incubated for 30 min. Subsequently, the cells were washed twice with PBS and harvested with 1X Laemmli buffer. Samples were separated on a 3-8% NuPAGE Tris-Acetate gel (Invitrogen, #EA0375BOX) and the fluorescent DNMT1 protein was visualized using a Typhoon imager (Amersham). The gel was subsequently stained with InstantBlue Coomassie Protein Stain (ISB1L) (Abcam, #ab119211) to visualize all proteins for normalization.

### Synchronization of cells to S phase and cell cycle analysis

To synchronize cells to G1/S phase of the cell cycle, a double thymidine block was performed. Briefly, cells were plated at 20% confluency and treated with 20 mM thymidine (Sigma-Aldrich, #T1895-1G) for 24 h, washed 3 times with phosphate-buffered saline (PBS) and allowed to recover for 12 h. Subsequently, 40 mM thymidine was added to cells for 24 h and cells were released from the block by washing with PBS. Cells were imaged and/or harvested at various timepoints after the second thymidine release. To determine if cells were synchronized to S phase, cells were stained with propidium iodide and analyzed by flow cytometry. Asynchronous or synchronized cells from 60 mm dishes were harvested and fixed in 66% ethanol. The fixed cells were washed with PBS and treated with 1:1000 RNase A (Thermo Fisher Scientific, #EN0531) for 5 min at 37°C. Next the cells were stained with 1X propidium iodide (Abcam, #ab139418) for 5 min at 37°C, washed with PBS and filtered. The filtered cells were analyzed with the Accuri flow cytometer (BD Biosciences) using the PE filter (ex 488 nm) and 5000 to 10000 events recorded. All data were processed and analyzed using FlowJo v10.

### Live cell single molecule imaging, single particle tracking and analysis

Live cell single molecule imaging was performed as described [37, 39] with these modifications. E8 (+/+) or F3 (+/-) cells were seeded to 45-60% confluency in 35 mm imaging dishes (Ibdi, USA, #81158). Cells were treated with 0.25 nM JF646 Halo ligand for 2 min and washed 5 times with DMEM. Alternatively, the cells were treated with 0.5 nM JF657 Halo ligand for 5 to 10 min and washed 3 times with DMEM. In all experiments, the Nikon Ti2-E epifluorescence microscope with an automated TIRF arm and two Andor Ixon 897 EMCCDs was used for image acquisition. To image fast moving DNMT1-Halo tagged molecules, cells were continuously illuminated with the 647 nm laser (35% laser power for JF646 ligand and 20% for JF657 ligand) for 10 s with an effective frame rate of ∼97 frames per second (fps), with a region of interest of 256 × 128 pixels; see Supplemental Movies SM1-16.

For single particle tracking analysis, the nuclei in every TIFF file were first cropped in Fiji/ImageJ. To generate trajectories of Halo-tagged DNMT1 molecules, the cropped nuclei were run through SLIMfast “ParallelProcess_fastSPT_JF646” (https://github.com/elifesciences-publications/SPT_LocAndTrack/tree/master) in MATLAB 2023a, with these settings: Localization Error = -5, Emission wavelength for JF646 = 664 or Emission wavelength for JF657 = 672, Exposure Time = 10, Deflation loops = 0, Max Expected Diffusion = 5, Number of gaps allowed = 2, NA = 1.49, PSF scaling = 1.35 and Pixel size = 0.16 µm. Next, these trajectories were run through Spot-on version 1.05 [42] to determine the diffusion coefficient and estimate the fraction of DNMT1 bound to chromatin, using a three state model (Supplementary Fig. S2A). The following parameters were used: TimeGap = 10, dZ = 0.700 μm, Gaps Allowed = 2, Time Points = 7, Bin Width = 0.01 μm, Jumps To Consider = 4, PDF-fitting, Number of States = 3, D_Free_2State = [0.5 10], D_Bound_2State = [0.0001 0.5], D_Free1_3State = [0.05 1], D_Free2_3State = [1 10], D_Bound_3State = [0.0001 0.05]. Each single molecule imaging experiment was conducted for at least 2-3 biological replicates with images acquired on different days with > 15 cells and > 30 cells for synchronized cells.

To visualize a subset of single molecule trajectories, such as the last 4.5 s of 10 s long TIFF file (∼ 45 fps), the cropped nuclei were run with “ParallelProcess_fastSPT_JF646” with these settings: Localization Error = -6, Emission wavelength = 664, Exposure Time = 22, Deflation loops = 0, Max Expected Diffusion = 4, Number of gaps allowed = 2, NA = 1.49, PSF scaling = 1.35 and Pixel size = 0.16 µm. The tracked files were run through “SPT Analysis” package (J. Schmidt, Michigan State University, USA) to generate plot for the DNMT1 trajectories in each cell.

### Chromatin fractionation assay following small molecule inhibition

For chromatin fractionation assay, 150 mm dishes of either E8 (+/+) or F3 (+/-) cells were incubated either with DMSO for 4 h, or 4 µM GSK or 5-azaC for 4, 24 or 48 h. 30 min prior to harvesting cells, cells were treated with 500 nM JF646 ligand to label the DNMT1-Halo-tagged protein. Cells were trypsinized, pelleted and washed 3 times with 1X PBS, and fractionated using the Pierce Subcellular Protein Fractionation kit (#78840, Thermo Fisher Scientific, USA) according to manufacturer’s instructions. For each sample, 250 µl of CEB or MEB buffer and 125 µl of NEB buffer were used from the kit. Subsequently, the amount of protein in the nucleoplasmic and chromatin fractions was quantified using Pierce BCA Protein Assay kit (#23227, Thermo Fisher Scientific) according to the manufacturer’s instructions, except that the nucleoplasmic fraction sample was diluted to 1:10 and only 10 µl of chromatin sample was used. 8 mg each of nucleoplasmic and chromatin fraction was separated on NuPAGE Tris-Acetate gel (Invitrogen, #EA0375BOX, USA), imaged with the Typhoon imager (Amersham) and stained with Coomassie blue dye as described above. Gel Analyzer v19.1 was used to quantify the level of DNMT1-Halo tagged protein and total protein levels (Coomassie blue staining). For immunoblotting, 5X sample buffer was added to 8 mg of either nucleoplasmic or chromatin fraction and all samples were prepared as described above.

## Results

### Generating Halo-tagged DNMT1 CRISPR-edited cell lines for live cell imaging

**Figure 1.**
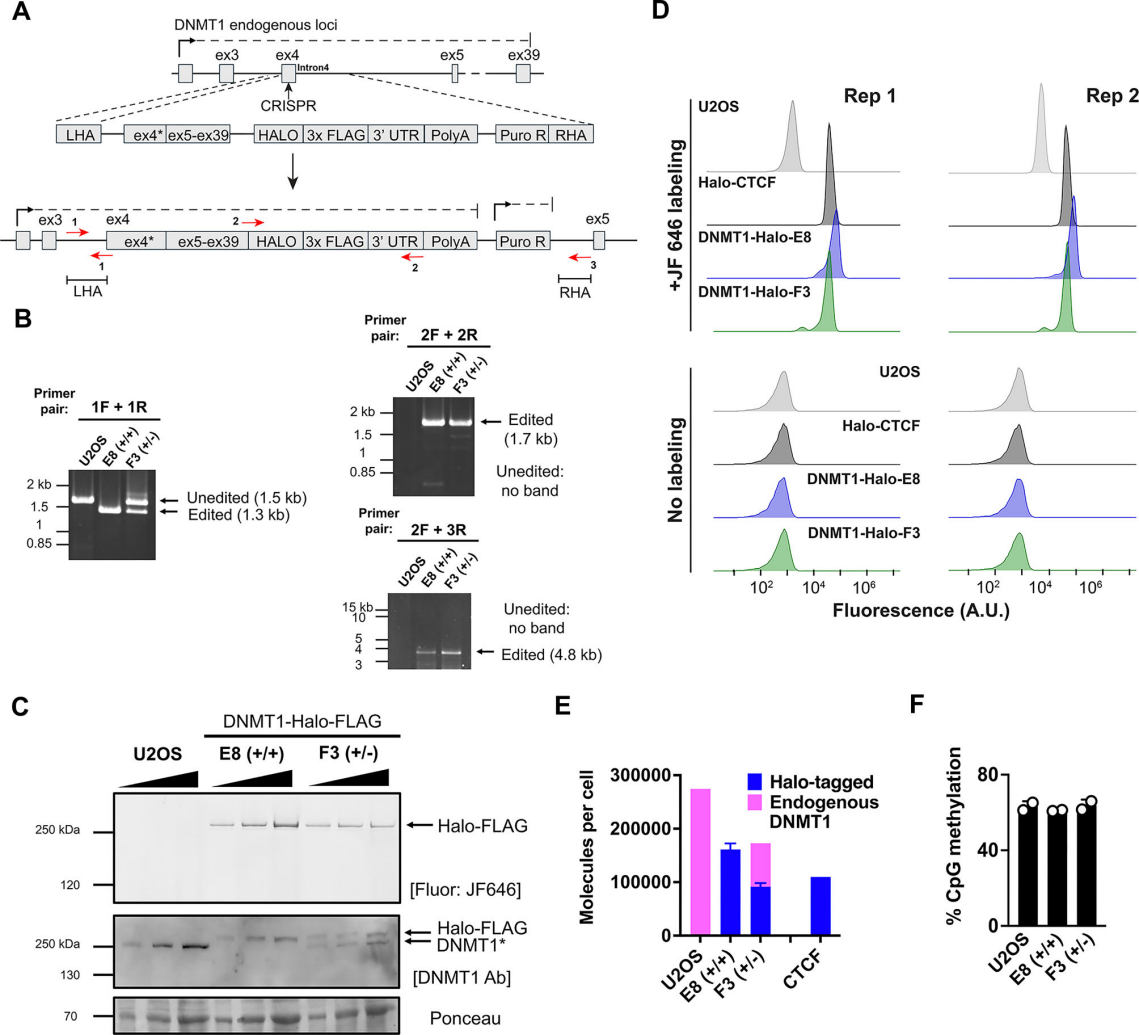
Genome-editing of Halo-tagged *DNMT1* and determining number of DNMT1 molecules per cell. (**A**) Schematic of CRISPR-editing for inserting sequences encoding the Halo-3XFLAG tag into the 3’ ends of *DNMT1* endogenous loci in U2OS cells. Red arrows, primer pairs used for PCR verification. (**B**) PCR verification of CRISPR-edited U2OS cell lines. gDNA was extracted from individual clones following puromycin selection and cell sorting of JF549-Halo dye-labelled cells. Primers are F = forward; R = reverse. (**C**) Top, 3–8% Tris-acetate gel stained with JF646 showing the presence of DNMT1-Halo-FLAG in the E8 (homozygous positive) and F3 (heterozygous) cell lines with parental U2OS cells as a control. Wedge, 2x serial dilutions. Middle, immunoblot showing the levels of endogenous (marked with *) and DNMT1 Halo-FLAG. The 70 kDa band on the Ponceau stain of the same blot was used as a loading control. (**D**) Flow cytometry estimating the abundance of Halo-tagged DNMT1 in E8 (+/+) and F3 (+/-) cell lines relative to Halo-CTCF in different U2OS cells. Two biological replicates are shown for both JF646-labeled and unlabeled cells. (**E**) Relative abundance of DNMT1-Halo and Halo-CTCF, endogenous (untagged) DNMT1 in the indicated cell lines, also see Supplementary Tables 1-3.**(F)** Percent methylation of CpG dinucleotides in genomic DNA determined by nanopore sequencing.

Preliminary experiments showed that DNMT1 with an *N*-terminal Halo-tag was unstable. Therefore, we generated human osteosarcoma (U2OS) cell lines expressing C-terminally tagged DNMT1 using CRISPR genome editing (Fig. 1A). The cDNA of *DNMT1* exons 3–39 fused to a Halo-3XFLAG tag was inserted into genomic loci of endogenous *DNMT1* on chromosome 19, of which there are two copies in most U2OS cell lines [43, 44]. To verify successful incorporation of sequences encoding the tag, we isolated genomic DNA (gDNA) from clones grown from single cells and amplified key regions using specific primers (as denoted in Fig. 1A). Amplification of primer set 1F and 1R (near the left homology arm), primer set 2F and 2R (near the Halo-3XFLAG tag) or primer set 2F and 3R (near the Halo-3XFLAG tag to right homology arm) showed that both alleles of *DNMT1* were Halo-tagged in clone E8 (homozygous positive (+/+)) whereas only one allele was tagged in clone F3 (heterozygous (+/-)), Fig. 1B.

After flooding the Halo-ligand conjugated to a red fluorophore (JF646) into U2OS cells, we visualized the DNMT1-Halo-FLAG protein in E8 (+/+) and F3 (+/-) cells but not in the parental U2OS cell line (Fig. 1C). The mass of the tagged protein was consistent with a protein containing full-length DNMT1 (∼183 kDa) plus the Halo-FLAG tag (∼34 kDa). Immunoblotting of DNMT1 showed that the steady-state level of tagged DNMT1 in the E8 (+/+) cells was reduced about 2-fold compared to parental cell line (Fig. 1C). This decrease in protein level could perhaps be due to reduced gene expression or lower stability of the Halo-tagged protein. The Halo-FLAG DNMT1 and endogenous DNMT1 were present at similar levels in the F3 (+/-) heterozygous cells. These experiments indicated that the DNMT1-Halo-FLAG protein was intact and present at levels within two-fold that of the endogenous protein.

To determine the number of molecules of Halo-tagged DNMT1 protein in the E8 (+/+) and F3 (+/-) cell lines, we compared their fluorescence intensities to those of cells containing a Halo-tagged CTCF standard using a flow cytometry approach previously described [39, 45]. We found that the level of Halo-tagged DNMT1 in E8 (+/+) cells (∼160,000 molecules per cell) was roughly double that of F3 (+/-) cells (∼ 90,000) (Fig. 1D–E and Supplementary Table 1-3). Using immunoblotting quantification estimates of DNMT1 levels in Fig. 1C, we were able to back calculate the amount of endogenous DNMT1 present in the parental cell line (∼275,000 molecules) and the endogenous DNMT1 level in the F3 (+/-) cell line (∼80,000 molecules) (Fig. 1E and Supplementary Table 3). In aggregate, these estimates are consistent with the expected DNMT1 protein levels in the parental U2OS and the genome-edited E8 (+/+) and F3 (+/-) cell lines.

Additionally, nanopore sequencing of various cell lines (see Supplementary Methods) revealed that the CpG DNA methylation levels in the Halo-tagged DNMT1 cell lines were similar to the parental U2OS cell line (∼ 65%, Fig. 1G). These results indicate that the Halo-tagged DNMT1 is functional *in vivo*.

### Single molecule tracking of DNMT1 in living cells

**Figure 2.**
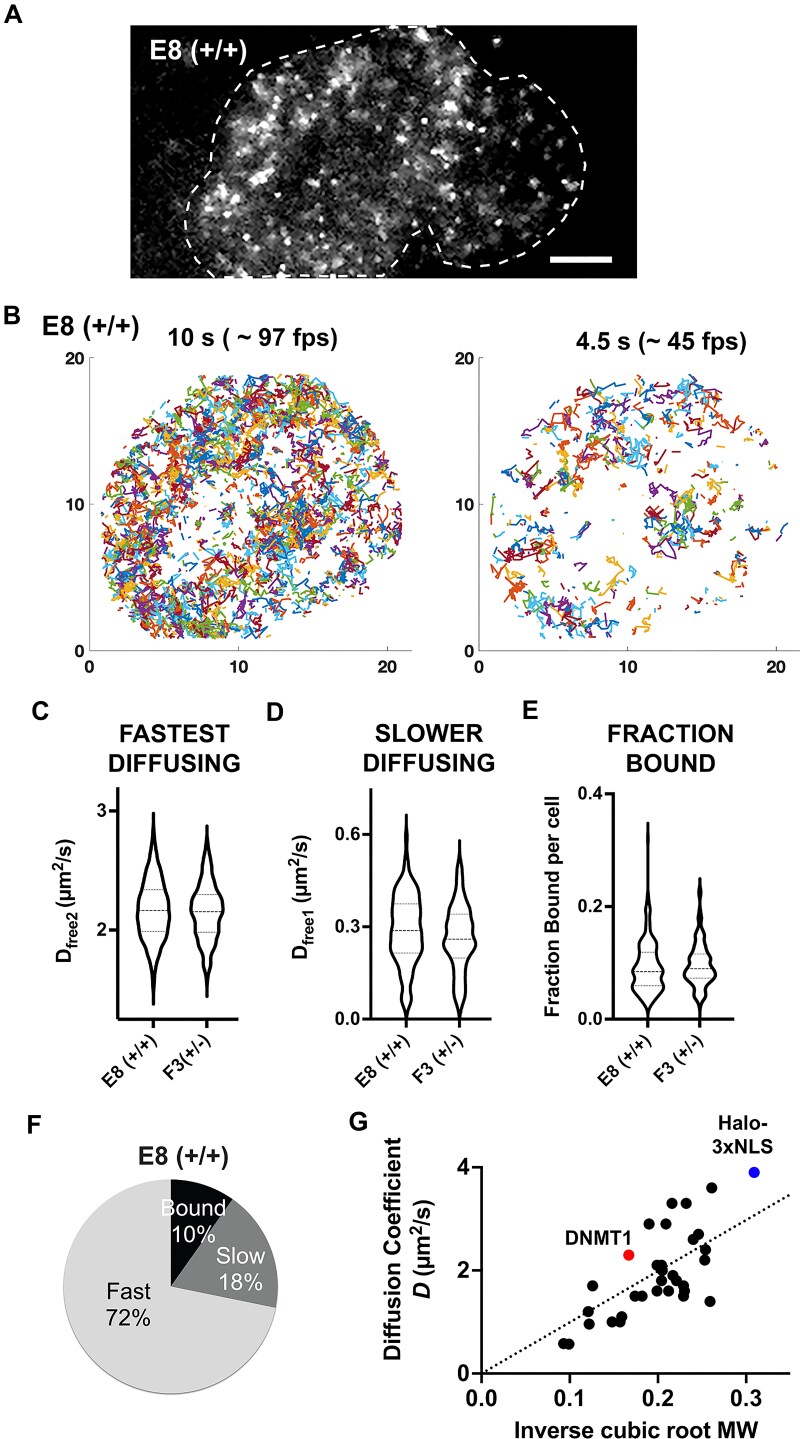
Tracking of DNMT1-Halo tagged molecules by live cell imaging. (**A**) Cell nucleus showing Halo-tagged DNMT1 molecules labeled with 0.25 nM JF646 Halo ligand for 5 min and imaged using HiLo microscopy at ∼ 97 fps over a 10 s period. Still from representative Halo-tagged DNMT1 E8 (+/+) movie (composite of five frames, ∼ 0.5 s). Dotted line denotes outline of the nucleus, scale bar = 5 µm. (**B**) Representative nucleus showing DNMT1-Halo-FLAG trajectories in an asynchronous E8 (+/+) cell. Left, all trajectories shown in a 10 s movie. Right, a subset of the trajectories from the last 4.5 s of the corresponding movie. (**C**-**F**) Spot-on analysis showing the dynamics of DNMT1-Halo-FLAG tagged molecules in the E8 (+/+) and F3 (+/-) cell lines, using a three-state model. **(C–E)** Violin plots showing the distribution of 183-236 cells across at least five replicates. **(F)** Pie chart showing the distribution of fast or slow diffusing and chromatin-bound DNMT1 molecules in asynchronous E8 (+/+) cells. **(G)** Diffusion coefficient (*D*) of various chromatin-modifying factors, transcription factors and DNA repair machinery proteins imaged by single molecule live cell imaging plotted against their molecular weight (represented as inverse cubic root molecular weight $\frac{1}{{\sqrt[3]{{MW}}}}$, units of *MW* = kDa). Dotted line represents the best fit (linear regression). Generally, the diffusion coefficient decreases with increased molecular weight of the protein. Source of data given in Supplementary Table 4.

By flooding the Halo-ligand JF646 into living U2OS cells, we visualized single DNMT1 molecules and studied their dynamics using HiLo microscopy (see Materials and Methods for details). As expected, the molecules were predominately nuclear, and DNMT1 diffused through most of the nuclear volume rather than being constrained to a particular compartment (Fig. 2A and Supplementary Fig. S1, Supplementary Movies 1 and 2). We determined single-molecule trajectories and analyzed them using the Spot-on analysis pipeline developed by the Tjian-Darzacq group [42]. A two-state model with fast-diffusing and bound components gave a poor fit to the data, but the addition of an intermediate “slower diffusing” component (D_free1_: 0.05 to 1 µm^2^/s) best described the trajectories of the DNMT1 molecules (Supplementary Fig. S1A). In asynchronous cells, ∼72% of DNMT1 was freely diffusing, another ∼18% was slower diffusing, and ∼10% had a diffusion coefficient consistent with being chromatin bound (Fig. 2F and Supplementary Fig. S1C). Representative live cell imaging movies of DNMT1 molecules in E8 (+/+) and F3 (+/-) cells are shown in Supplementary Movies 1 and 2.

Next, we plotted the average diffusion coefficient of free DNMT1 molecules (D_free1_ and D_free2_) and the fraction of bound DNMT1 molecules for each cell (Fig. 2C–E), estimated from ∼ 5000 to 10,000 trajectories tracked per cell (representative trajectories are shown in Fig. 2B and Supplementary Fig. S1D). Note that each violin plot represents the average calculated diffusion coefficient or fraction of DNMT1 bound per cell, and we plotted these values from 183 to 236 cells and across at least 5 biological replicates (Fig. 2C–E). The diffusion coefficient of the fastest DNMT1 diffusing molecules was as expected for a protein of this size, given the Stokes-Einstein relationship and the diffusion coefficients observed for other chromatin modifying factors, transcription factors and DNA damage factors (Fig. 2G and Supplementary Table 4). The intermediate diffusing population might represent DNMT1 bound in a multi-protein complex or bound to RNA, given that DNMT1 has a higher affinity for RNA than for nucleosome-linker sized DNA *in vitro* [46].

### DNMT1 is less mobile and more chromatin-bound during S phase of the cell cycle

**Figure 3.**
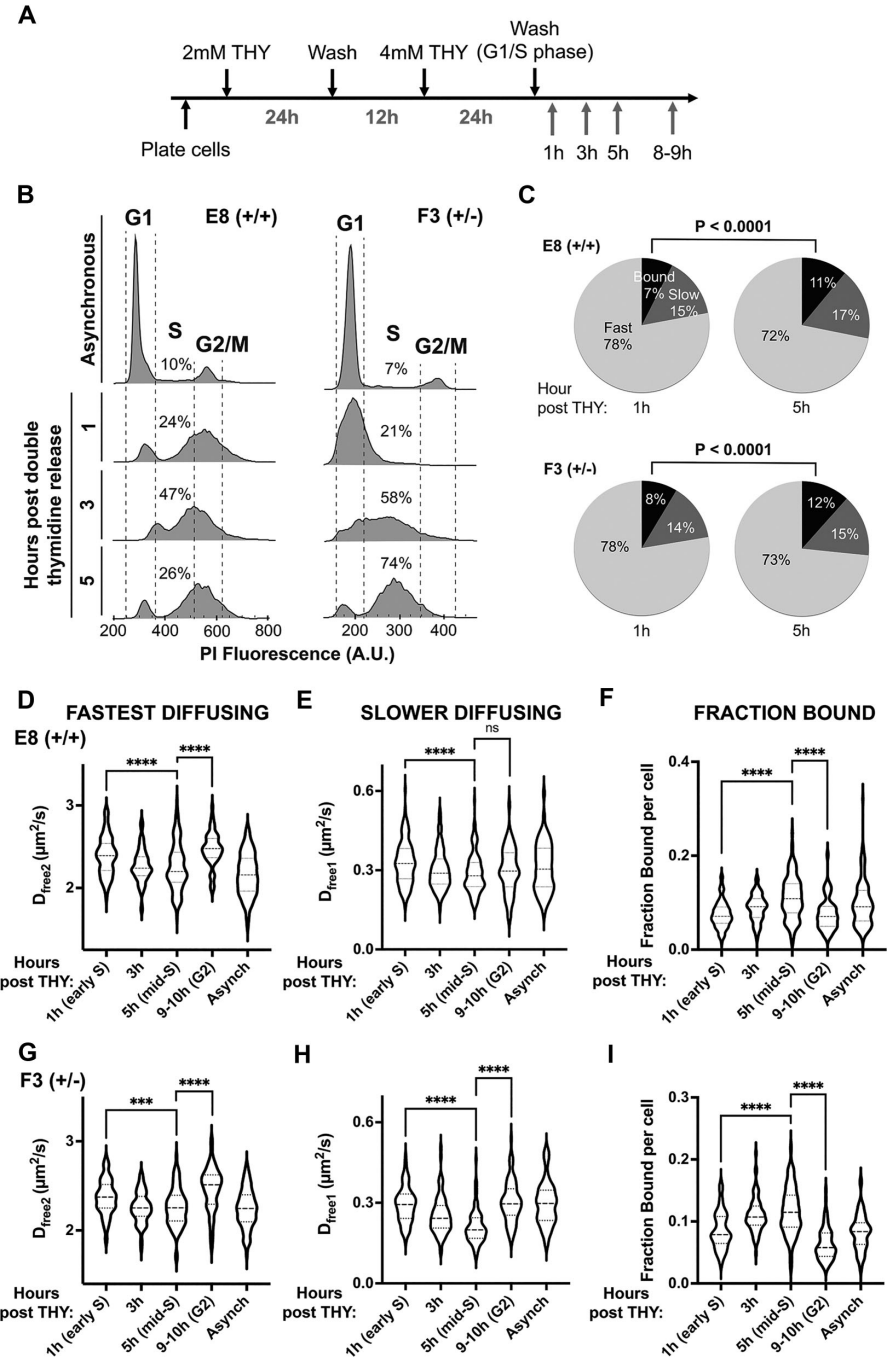
Dynamics of DNMT1 through the S phase of the cell cycle. (**A**) Double thymidine block protocol used to synchronize cells to S phase. (**B**) Flow cytometry after propidium iodide staining to show cell cycle progression (based on the DNA content of the cells). Following 1 h post-thymidine release, there is an increase in S phase cells which culminates at 3–5 h post-release. (**C**-**I**) Cells were synchronized using a double thymidine block and labelled as described in text. Spot-on analysis showing the dynamics of DNMT1-Halo molecules. **(C)** Pie charts showing the distribution of fast or slow diffusing and chromatin-bound DNMT1 molecules at 1 and 5 h post-thymidine release. **(D-I)** Violin plots showing the distribution of DNMT1-Halo in 62-152 cells across 2–5 replicates. **(D and E)** and **(G and H)** For fastest and slower diffusing molecules, mid-S phase cells (5 h post-thymidine release) have less mobile DNMT1-Halo compared to cells at G1/S (1 h). **(F)** and **(I)** The fraction of DNMT1-Halo tagged molecules that are chromatin bound increases in mid-S phase (5 h) compared to G1/S (1 h) and G2 phase (9–10 h) of the cell cycle. Student t-test, two-tailed, **** *P* < 0.0001, *** *P* < . 0.001, ns = not significant.

During the S phase of the cell cycle, DNMT1 is associated with the DNA replication machinery and methylates newly synthesized DNA. Previous reports using FRAP and live cell imaging of bulk fluorescently tagged DNMT1 suggested that DNMT1 is less mobile in S phase [13, 47]. To study the dynamics of individual molecules of DNMT1 in live cells, we synchronized parental U2OS, E8 (+/+) or F3 (+/-) cell lines using a double thymidine block protocol (Fig. 3A and Supplementary Fig. S2A-B). To verify the cell cycle synchronization, we performed propidium iodide staining followed by flow cytometry. At 3-5 h post-thymidine release, the number of cells in S phase was enriched compared to asynchronous cells (Fig. 3B and Supplementary Fig. S2B, 47% in S for E8 (+/+) and 58% for F3 (+/-) cells 3 h post-thymidine release). For the parental U2OS cell line, the number of S phase cells was most enriched at 1 h post- thymidine release, but the level was comparable to that of the Halo-tagged DNMT1 cell lines at 3 h post-release (Supplementary Fig. S2A-B).

In S phase synchronized cells, we observed a modest but reproducible decrease in the diffusion coefficient for both fast (D_free2_) and slower (D_free1_) diffusing DNMT1 molecules, which peaked at 5 h post-thymidine release (mid-S phase) (Supplementary Movie 3 and 4, Fig. 3C–E, 3G–H, D_free2 _= 2.39 µm^2^/s for 1 h vs 2.26 µm^2^/s for 5 h in E8 (+/+) cells, D_free2_ = 2.37 µm^2^/s for 1 h vs 2.26 µm^2^/s in F3 (±) cells; D_free1_ = 0.39 µm^2^/s for 1 h vs 0.29 µm^2^/s for 5 h in E8 (+/+) cells, D_free1 _= 0.29 µm^2^/s for 1 h vs 0.21 µm^2^/s in F3 (+/-) cells). This decrease correlates with a significant increase in the fraction of DNMT1 bound to chromatin (Fig. 3C, F and I). These changes can also be seen in the particle tracking histograms, which show that the jump length of DNMT1 molecules decreases in mid-S phase cells (compare 1 h and 5 h post-thymidine release) (Supplementary Fig. S2C-D). Collectively, these results show that a modest fraction of DNMT1 (∼12%) is localized to chromatin during the S phase of the cell cycle, presumably bound to the DNA replication machinery.

### DNMT1 is more dynamic during the G2 phase of the cell cycle

Next, we tested if the dynamics of DNMT1 change in other phases of the cell cycle. Previous studies employing overexpression of GFP-tagged DNMT1 suggested that DNMT1 became more associated with chromatin during G2 phase [13]. To determine whether DNMT1 protein dynamics are altered in G2, we synchronized E8 (+/+) and F3 (+/-) cells using the Cdk1 inhibitor RO-3306, which arrests cells at G2/M phase (Supplementary Fig. S3A). One method to distinguish cells in G2/M from other phases of the cell cycle is to observe the microtubule organizing center (MTOC). The MTOC is duplicated during S phase, and one MTOC migrates to the opposite side of the nucleus in G2 in preparation for cell division [48]. In asynchronous cells, immunofluorescence staining of the MTOC (γ-tubulin staining) showed that most cells had one MTOC, whereas 1 h post-RO release, a greater proportion of cells had 2 MTOCs on opposite sides of the nucleus and had the characteristic mitotic spindle staining (Supplementary Fig. S3B). These results confirmed the successful synchronization of the majority of cells to G2/M phase.

Using live cell single molecule imaging, we unexpectedly found that cells synchronized to G2 (1 h post-RO release) had an increased diffusion coefficient for the fastest diffusing (D_free2_) molecules, compared to asynchronous cells (Supplementary Movie 5 and 6, Supplementary Fig. S4A-C, left panels of Supplementary Fig. S4D and G, D_free2 _= 2.47 µm^2^/s for 1 h post RO release vs 2.17 µm^2^/s for asynchronous E8 (+/+) cells; D_free2 _= 2.60 µm^2^/s for 1 h vs 2.06 µm^2^/s for asynchronous F3 (+/-) cells). Note that the majority of asynchronous cells are in the G1 phase as determined by flow cytometry followed by propidium iodide staining (Fig. 3B and Supplementary Fig. S2A). This increase in diffusion coefficient in the G2 phase was accompanied by a decrease in fraction bound of DNMT1 molecules (Supplementary Fig. S4A, left panels of Supplementary Fig. S4F and I) which was especially pronounced for the F3 (+/-) cells. We then compared RO-3306 synchronized cells to the equivalent timepoint in cells synchronized using a double thymidine block (9-10 h post thymidine release) and observed similar results (Supplementary Fig. S4D-I, right panels, D_free2 _= 2.26 µm^2^/s for 5 h vs 2.47 µm^2^/s for 9-10 h E8 (+/+) cells; D_free2 _= 2.26 µm^2^/s for 5 h vs 2.47 µm^2^/s for 9-10 h F3 (+/-) cells). Our results suggest that (at least in our U2OS genome-edited cell lines) DNMT1 molecules that are ‘tethered’ to the DNA replication machinery in S phase are released in G2 phase and become more mobile prior to mitosis. Why this differs from a previous report will be explored in the Discussion section.

### The disordered *N*-terminal domain of DNMT1 influences its mobility

**Figure 4.**
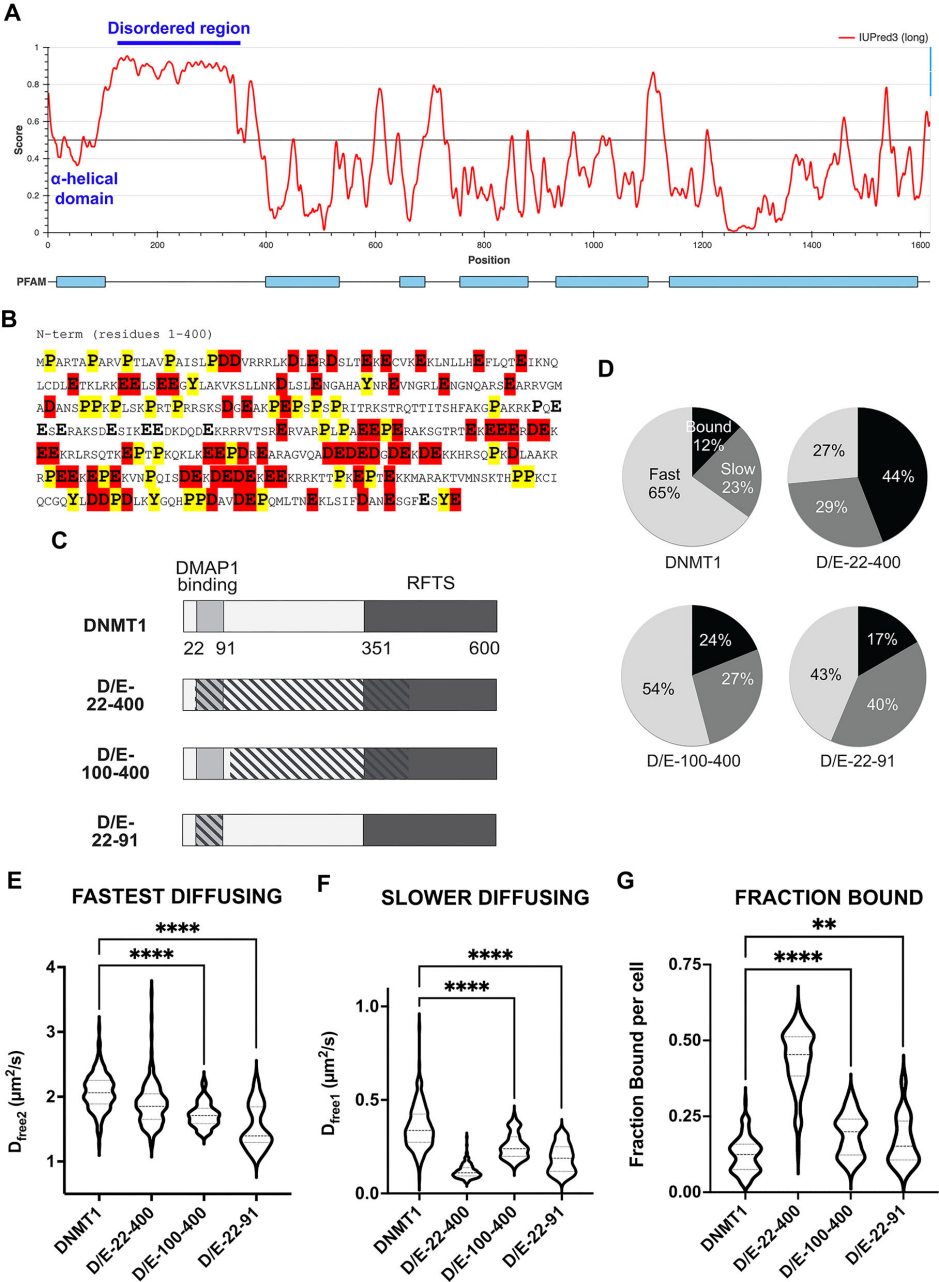
DNMT1 *N*-terminal mutants have decreased DNMT1 dynamics. (**A**) IUPred3 of DNMT1 showing the disordered domain (residues 100-400) and α-helical domain (residues 22-91). (**B**) *N*-terminal sequence of DNMT1, all Asp (A) and Glu (E) residues are highlighted in red, Pro (P) and Tyr (Y) are highlighted in yellow. Only the first 400 amino acids are shown. (**C**) Schematic of the DNMT1 *N*-terminal mutants, all D and E residues in the hatched region were mutated to alanine. Only the first 600 amino acids are shown. (**D–G**) Wildtype DNMT1 and *N*-terminal mutants were transfected into U2OS cells, and 18-24 h later live cell single molecule imaging was performed. Spot-on analysis showing the dynamics of DNMT1-Halo molecules. **(D)** Pie charts showing the distribution of fast or slow diffusing and chromatin-bound DNMT1 of various *N*-terminal mutants. **(E–G)** Violin plots showing the distribution of DNMT1-Halo in 17 to 214 cells across 2–5 replicates. All *N*-terminal DNMT1 mutants are less mobile compared to WT DNMT1. **(G)** The fraction bound of D/E-1-400 mutant is significantly increased compared to DNMT1 and other mutants. Student t-test, two tailed, **** *P* < 0.0001, ** *P* < 0.01.

Previous work from others suggests that the *N*-terminal region of DNMT1 regulates its function as a DNA methyltransferase or transcriptional repressor [19, 23]. Therefore, we wondered if mutations in this region influence DNMT1 mobility. Strikingly, the *N*-terminal region contains an IDR that is enriched for glutamic acid (Glu), aspartic acid (Asp), proline (Pro) and tyrosine (Tyr) residues (Fig. 4A–B). Moreover, a recent study identified an α helical domain (residues 22 to 91) that binds to DMAP1 and is required for transcriptional repression [20, 21]. Based on this, we generated mutants where all the Asp and Glu residues were mutated to alanine in either the DMAP1 binding region (D/E-22-91), the IDR alone (D/E-100-400) or in both (D/E-22-400) (Fig. 4C). Subsequently, we transiently transfected these mutants in U2OS cells and performed live cell single molecule imaging as described above. The use of transient transfection for imaging these mutants was justified by our observations that transient transfection of wildtype Halo-tagged DNMT1 gave similar single molecule tracking results to those of CRISPR-edited knock-in Halo-tagged DNMT1 in the E8 (+/+) and F3 (+/-) cell lines (compare Fig. 2C–F and Supplementary Fig. S1C to Fig. 4D–G).

Furthermore, mutations in the IDR domain decreased the DNMT1 diffusion coefficient (Supplementary Movie SM7 to 10, Fig. 4D–F, D_free2_ = 1.72 µm^2^/s for D/E-100-400 vs 2.07 µm^2^/s for wildtype, D_free1_ = 0.25 µm^2^/s for D/E-100-400 vs 0.35 µm^2^/s for wildtype) and increased the fraction of DNMT1 bound to chromatin (Fig. 4G, 19% fraction bound for D/E-100-400 compared to 12% for wildtype DNMT1). Mutations in the entire *N*-terminal domain (D/E-22-400) dramatically increased DNMT1 binding to chromatin by four fold (44% fraction bound for D/E-22-400 compared to 12% for wildtype DNMT1). Based on our results, it is conceivable that the aspartic and glutamic acid residues within the IDR of DNMT1 facilitate its relocalization from chromatin to the nucleoplasm.

Mutations localized to the region of DNMT1 that binds to DMAP1 (D/E-22-91) greatly influenced DNMT1 dynamics and sub-nuclear localization. The D/E-22-91 mutant had decreased diffusion rate (Fig. 4D–F, D_free2_ = 1.54 µm^2^/s for D/E-22-91 vs 2.07 µm^2^/s for wildtype, D_free1_ = 0.19 µm^2^/s for D/E-22-91 vs 0.25 µm^2^/s for wildtype) and increased binding to chromatin (from 12% to 17%, Fig. 4G). Our results suggest that mutations in this region stabilize DNMT1 onto chromatin, preventing the mutant DNMT1 from dissociating into the nucleoplasm. How the extreme *N*-terminal region of DNMT1 alters DNMT1 dynamics and in turn influences its function will be explored in the Discussion.

### Acute treatment with GSK significantly decreases the mobility of DNMT1

**Figure 5.**
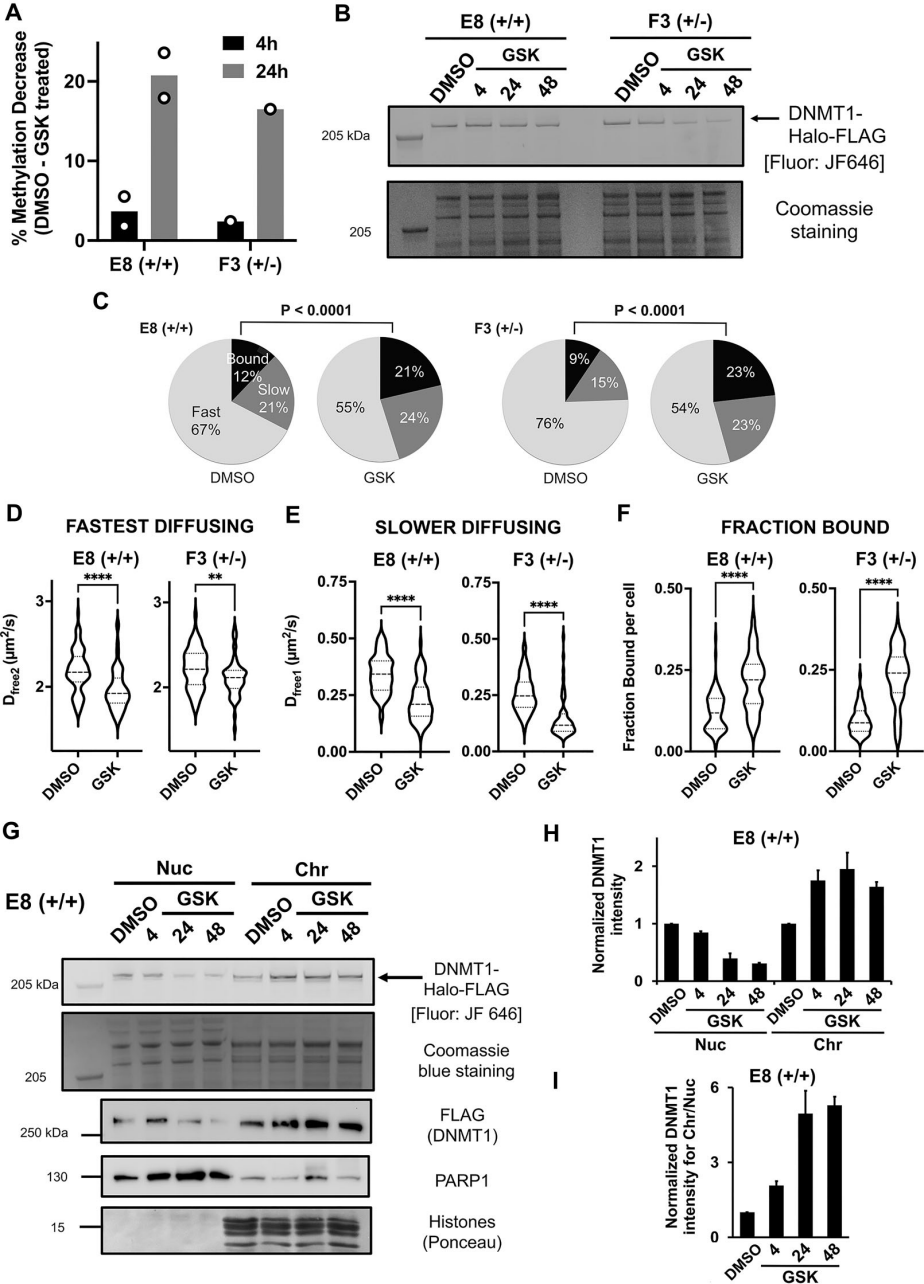
Acute treatment with GSK significantly decreases DNMT1 mobility. (**A**) Decreased CpG methylation levels in cells treated with the DNMT1-specific inhibitor GSK-3484862 (GSK). Cells were treated with 4 μM GSK for 4 or 24 h, then gDNA was extracted from these samples and subjected to nanopore sequencing. The % methylation difference between the DMSO- and GSK-treated cells is shown. (**B**) Cells were treated with DMSO or 4 μM GSK for 4, 24, or 48 h, harvested using Laemmli buffer and proteins separated on a 3–8% Tris-Acetate gel. DNMT1-Halo tagged protein was visualized by incubating cells with 500 nM JF646 Halo-ligand for 30 min before harvesting. Coomassie staining of ∼ 205 kDa region is shown as a loading control. (**C**-**F**) Cells were treated with either DMSO or 4 μM GSK for 4 h. Images were acquired using HiLo microscopy at ∼ 97 fps over a 10 s period with no delay. Spot-on analysis: **(C)** Pie chart distribution of the DNMT1 molecules following either DMSO or GSK treatment for 4 h. **(D-F)** Effect of 4 h GSK treatment on DNMT1 diffusion and chromatin binding. Violin plots show the distribution of DNMT1-Halo in 73–87 cells across 4 replicates. Student t-test, two-tailed, **** *P* < 0.0001, ** *P* < 0.01, ns = not significant. **(G-I)** GSK treatment relocalizes DNMT1 from the nucleoplasm to chromatin. **(G)** E8 (+/+) cells treated with DMSO for 4 h or with GSK for 4, 24, or 48 h were fractionated into nucleoplasm and chromatin fractions and DNMT1-Halo protein analyzed by JF646 fluorescent gel or immunoblot against FLAG. Note that the levels of DNMT1 in the nucleoplasm decrease following GSK treatment, consistent with total levels shown in (B). PARP1 and histones (from Ponceau gel) are used as protein markers for each compartment. **(H)** Relative DNMT1 intensity, normalized to total protein loading, from JF646 fluorescent gel. Error bars represent standard error of the mean between 2 and 3 replicates, see Supplementary Fig. S7A and B. **(I)** DNMT1 intensity in the chromatin fraction divided by that in the nucleoplasmic fraction. Error bars represent standard error of the mean.

GSK-3484862 (GSK) is a non-nucleoside, non-covalent small molecule inhibitor of DNMT1. Previous studies showed that long term treatment with GSK (>24 h) decreased global DNA methylation and caused DNMT1 protein degradation in multiple cell lines [32]. In agreement with these findings, we found that 24 h treatment of E8 (+/+) and F3 (+/-) cell lines with 4 µM GSK reduced 5-methyl-cytosine levels by ∼20% (Fig. 5A and Supplementary Fig. S5A). Compare this to complete inhibition of the enzyme, which would decrease 5-methyl-cystosine levels by 50% per population doubling. Consistent with previous reports, the protein levels of Halo-FLAG-tagged DNMT1 decreased following long-term treatment (>24 h) of E8 (+/+) and F3 (+/-) cell lines (Fig. 5B and Supplementary Fig. S5B). Similarly, the levels of endogenous untagged DNMT1 decreased following long term GSK treatment (> 24 h) in both parental U2OS and F3 (+/-) cell lines (Supplementary Fig. S5C).

Next, we tested if GSK treatment affected the subcellular localization of DNMT1. This was of interest because proteasomal degradation normally occurs in the cytoplasm and certain small molecule inhibitors perturb the localization of their target proteins [49]. GSK inhibition did not influence Halo-tagged DNMT1 nuclear localization (even after 72 h) but decreased the level of fluorescent DNMT1 protein (Supplementary Fig. S5D). Interestingly, GSK treatment triggered formation of punctate DNMT1 subnuclear structures in E8 (+/+) cells (Supplementary Fig. S5D-E, compare box I & II).

To determine whether GSK perturbs DNMT1 dynamics before the chromatin environment is altered by drug-induced DNA hypomethylation, we treated our Halo-tagged DNMT1 cell lines with either DMSO or 4 µM GSK for 4 h and performed live cell single molecule imaging. We confirmed that DNA methylation levels were minimally affected at this short time (Fig. 5A and Supplementary Fig. S5A). Following acute GSK treatment, the diffusion coefficient for both fast and slow moving DNMT1 molecules significantly decreased compared to DMSO-treated cells (Supplementary Movie 11 and 12, Fig. 5C–E; in E8 (+/+) cells, D_free2 _= 1.9 µm^2^/s for GSK-treated compared to 2.2 µm^2^/s for DMSO-treated, D_free1_ = 0.24 µm^2^/s for GSK-treated versus 0.38 µm^2^/s for DMSO-treated). This decrease was accompanied by a two-fold increase in the fraction of DNMT1 molecules bound to chromatin (Fig. 5C and F; in E8 (+/+) cells, 21% chromatin bound for GSK-treated cells versus 12% DMSO-treated), a much larger change than previously observed upon entry into S-phase. Similarly, displacement histograms showed a dramatic decrease in jump length following GSK treatment (Supplementary Fig. S6A-B). Collectively, these findings demonstrate that acute GSK treatment dramatically decreases DNMT1 dynamics, preceding inhibition of DNA methylation or DNMT1 degradation. [Note that GSK inhibition of DNMT1 produced minimal changes in the cell cycle for either cell line (Supplementary Fig. S5F).]

We next tested if DNMT1 relocalizes to the chromatin fraction following GSK treatment using a biochemical method orthogonal to single molecule imaging. E8 (+/+) or F3 (+/-) cells that were either DMSO- or GSK-treated were fractionated into nucleoplasmic and chromatin fractions, and the levels of DNMT1 were assessed. Protein markers confirmed that the fractionation was successful; PARP1 as a nucleoplasmic marker and histones as a chromatin marker (Fig. 5G and Supplementary Fig. S6C). Following GSK treatment, Halo-FLAG-tagged DNMT1 levels decreased in the nucleoplasm and increased in the chromatin fraction across three biological replicates (Fig. 5G–I, Supplementary Fig. S6C–E and Supplementary Fig. S7). Quantification of DNMT1 intensity (using the JF646 signal), normalized against total protein levels, showed enrichment of DNMT1 onto chromatin following GSK treatment at the 24 h timepoint (Fig. 5H–I and Supplementary Fig. S6D-E). The decrease in nucleoplasmic DNMT1 was consistent with the decrease in steady state DNMT1 levels as observed for whole cell lysate preparations (Fig. 5B). Taken together, these results confirm that GSK causes DNMT1 to relocalize from the nucleoplasm to chromatin, as discussed further below.

### Acute treatment with 5-azaC decreases the mobility of DNMT1 in S phase

**Figure 6.**
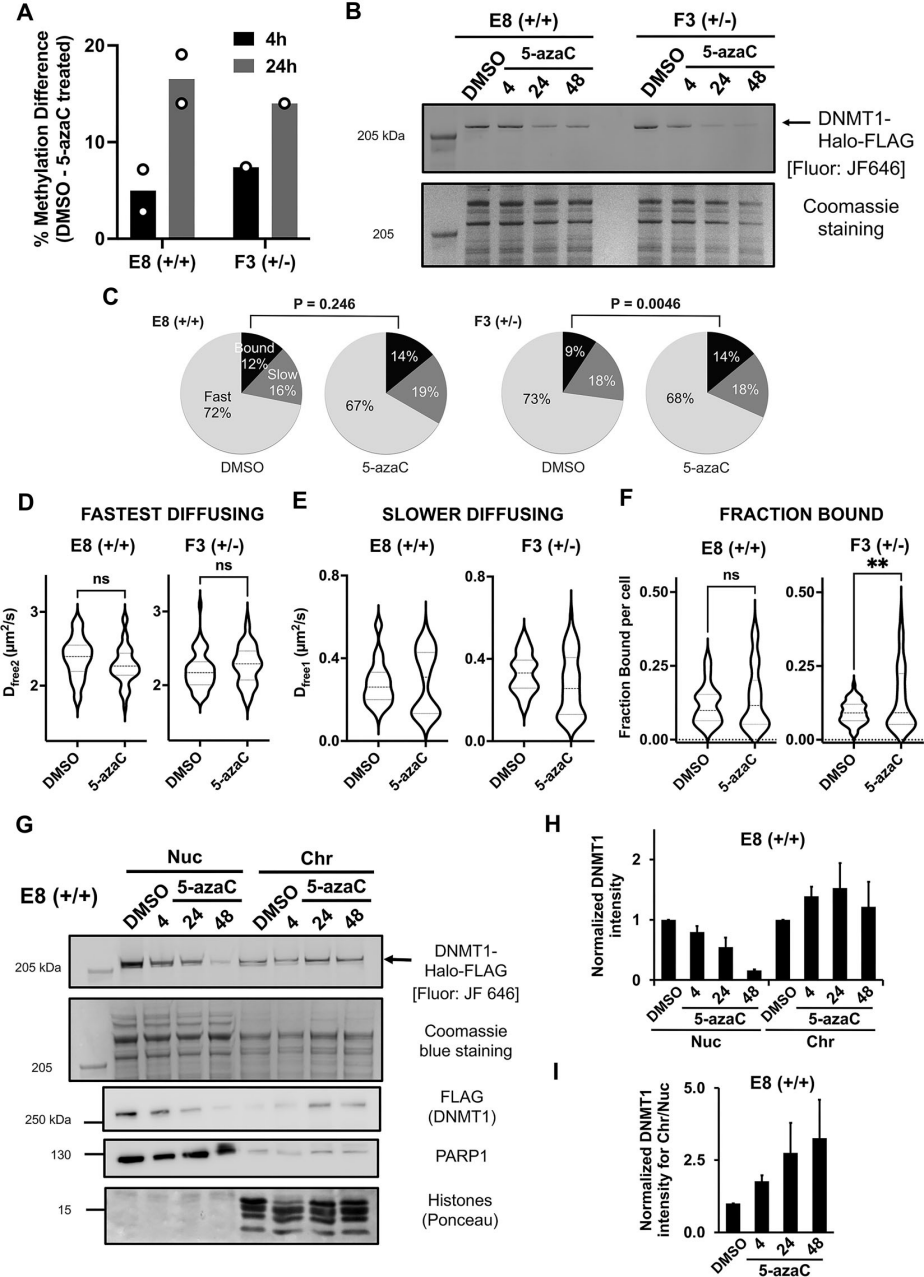
Acute treatment with 5-azaC decreases the mobility of a subset of DNMT1. (**A**) CpG methylation levels in cells treated with 5-azaC. Cells were treated with 4 μM 5-azaC for 4 or 24 h, gDNA was extracted and subjected to nanopore sequencing. The % methylation difference between the DMSO- and 5-azaC-treated cells is shown. (**B**) Cells were treated with DMSO for 4 h or 4 μM 5-azaC for 4, 24 or 48 h, harvested using Laemmli buffer and proteins separated on a 3–8% Tris-acetate gel. DNMT1-Halo protein was visualized by incubating cells with 500 nM JF646 Halo-ligand for 30 min before harvesting. Coomassie staining of ∼ 205 kDa region is shown as a loading control. (**C–F**) Cells were treated with either DMSO or 4 μM 5-azaC for 4 h. Images were acquired using HiLo microscopy at ∼ 97 fps over a 10 s period with no delay. **(D-F)** Spot-on analysis. Following 5-azaC treatment, the mobility of ∼ 50% of the slower diffusing DNMT1 molecules was significantly decreased, corresponding to an increase in the bound fraction of DNMT1. Violin plots showing the distribution of DNMT1-Halo 32–40 cells across two replicates. Student t-test, two-tailed, ** *P* < 0.01, ns = not significant. (G-I) 5-azaC treatment relocalizes DNMT1 from the nucleoplasm to chromatin. (**G**) E8 (+/+) cells treated with either DMSO or 5-azaC were fractionated into nucleoplasm and chromatin fractions. DNMT1-Halo protein is shown by JF646 fluorescent gel or immunoblot against FLAG. Note that the level of DNMT1 in the nucleoplasm decreases following 5-azaC treatment, consistent with total levels shown in (B). PARP1 and histones (from Ponceau gel) are used as protein markers for each compartment. (**H**) Relative DNMT1 intensity, normalized to total protein loading, from JF646 fluorescent gel, error bars represent standard error of the mean between three replicates, see Supplementary Fig. S10A and B. **(I)** DNMT1 intensity in the chromatin fraction divided by the nucleoplasmic fraction, error bars represent standard error of the mean.

Another small molecule DNMT1 inhibitor that is used clinically is 5-azaC. 5-azaC is a cytidine analog with a nitrogen atom at the position of the base normally methylated by DNMT1. 5-azaC is converted intracellularly into 5-aza-deoxyCTP, incorporated into DNA, and covalently traps DNMT1, which leads to loss of DNA methylation and targets chromatin-bound DNMT1 for degradation [30, 50, 51]. Consistent with this expectation, we found that 24 h treatment of either E3 (+/+) or F3 (+/-) cell lines with 4 µM 5-azaC led to ∼13% loss in DNA methylation (Fig. 6A and Supplementary Fig. S8A). Similarly, prolonged 5-azaC treatment led to degradation of Halo-FLAG-tagged and endogenous DNMT1 in both E8 (+/+), F3 (+/-) and parental U2OS cells, respectively (Fig. 6B and Supplementary Fig. S8B-C).

5-azaC treatment did not perturb Halo-tagged DNMT1 nuclear localization, but it decreased the fluorescence level of Halo-tagged DNMT1 as expected (Supplementary Fig. S8D). Interestingly, a 4 h 5-azaC treatment (at which point there was only about 5% decrease in methylated DNA (Fig. 6A) triggered the formation of punctate DNMT1 subnuclear structures in E8 (+/+) cell line (Supplementary Fig. S8D and E, see box I). Intriguingly, the trapping of DNMT1 in these punctate structures may explain the decreased DNMT1 dynamics and increased chromatin binding as described below.

To determine whether 5-azaC influences DNMT1 mobility, we treated our Halo-tagged DNMT1 cell lines with either DMSO or 4 µM 5-azaC for 4 h and performed live cell single molecule imaging. Following 5-azaC treatment, two populations of cells were observed, one with DNMT1 dynamics similar to the DMSO-treated cells and another with slower moving DNMT1 molecules. Quantification showed that the diffusion coefficients of slow diffusing (D_free1_) DNMT1 molecules became bimodal, with about half of the cells showing slow diffusion (Supplementary Movie 13 and 14, Fig. 6E). Note that the change in diffusion coefficient was accompanied by an increase in the fraction of DNMT1 molecules bound to chromatin (Fig. 6F). These changes in dynamics are reflected in the displacement histograms shown in Supplementary Fig. S9A and B. Collectively, these results suggest that 5-azaC acts on a subset of Halo-tagged DNMT1 cells (∼ 35-50% of cells imaged).

**Figure 7.**
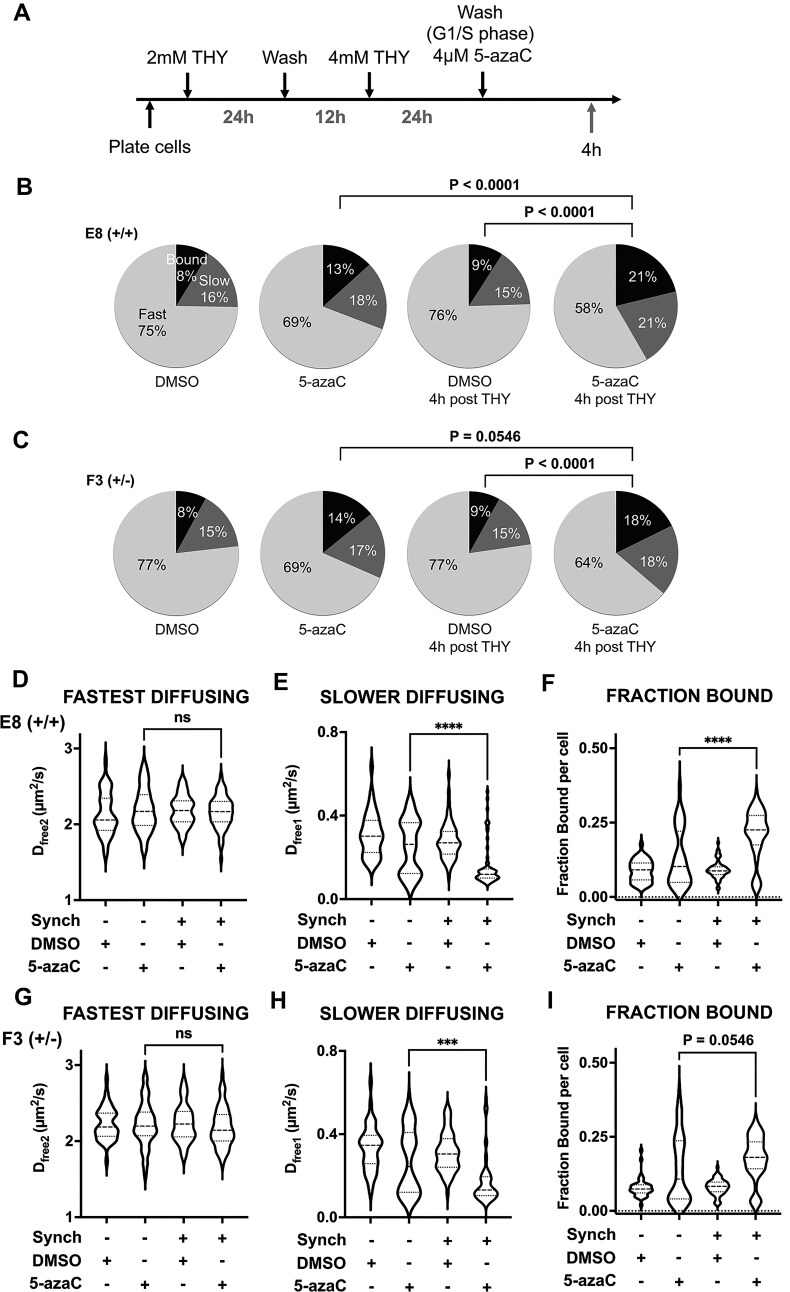
5-azaC inhibition of DNMT1 dynamics occurs in the S phase of the cell cycle. (**A**) Schematic of double thymidine block protocol used to synchronize cells, followed by either DMSO or 4 μM 5-azaC treatment for 4 h. (**B–I**) Spot-on analysis of cells synchronized to S phase and treated with 5-azaC. (B–C) Pie charts showing the distribution of DNMT1 molecules following DMSO and 5-azaC treatment in either asynchronous or synchronized (4 h post-THY) cells. **(D–I)** Violin plots showing the diffusion coefficients and distribution of DNMT1-Halo in 40–65 cells across three replicates. Student t-test, two-tailed, **** *P* < 0.0001, ns = not significant.

For 5-azaC to inhibit DNMT1, this nucleoside analog must be first incorporated into the DNA, which can only occur during the S phase of the cell cycle. Therefore, we hypothesized that 5-azaC inhibition of DNMT1 would be cell cycle dependent. To test this idea, we first synchronized E8 (+/+) or F3 (+/-) cells to the G1/S phase using a double thymidine block, then treated the synchronized cells with either DMSO or 5-azaC for 4 h (Fig. 7A). We found that the diffusion coefficient of slow moving DNMT1 molecules (D_free1_) was dramatically decreased in 5-azaC S-phase synchronized cells (Fig. 7E and H, D_free1 _= 0.16 µm^2^/s for 5-azaC treated synchronized E8 (+/+) cells vs 0.25 µm^2^/s for asynchronous cells; D_free1 _= 0.17 µm^2^/s for 5-azaC-treated synchronized F3 (+/-) cells vs 0.27 µm^2^/s for asynchronous cells). This decrease was accompanied by a greatly increased fraction of DNMT1 molecules bound to chromatin (Fig. 7B–C, F and I). In short, the bimodal distributions of DNMT1 dynamics caused by 5-azaC can be explained by cell cycle effects, with the slow-diffusing and higher fraction-bound molecules residing in S-phase cells. Importantly, using propidium iodide stained E8 (+/+) or F3 (+/-) cells, we found that 5-azaC drug itself had minimal effects on the cell cycle status (Supplementary Fig. S8F), despite having a cell cycle dependence for its mechanism of inhibition.

Orthogonal to the live cell imaging approach, we tested biochemically if DNMT1 relocalized to the chromatin fraction post-5-azaC treatment. E8 (+/+) or F3 (+/-) cells that were either DMSO- or 5-azaC treated were fractionated into nucleoplasmic and chromatin fractions, and the levels of DNMT1 were assessed. Following 5-azaC treatment, Halo-FLAG-tagged DNMT1 levels decreased in the nucleoplasm and increased in the chromatin fraction across 3 to 4 biological replicates (Fig. 6G–I, Supplementary Fig. S9C-E and Supplementary Fig. S10). Quantification of DNMT1 intensity showed enrichment of DNMT1 onto chromatin following 5-azaC treatment (Fig. 6H–I and Supplementary Fig. S9D-E). The decrease in nucleoplasmic DNMT1 was consistent with the decrease in total DNMT1 levels as observed for whole cell lysate preparations (Fig. 6B).

Because cells convert 5-azaC to 5-aza-deoxyC prior to incorporation into DNA, we predicted that the latter compound (generic name decitabine) would cause similar changes in DNMT1 dynamics. Indeed, decitabine decreased the diffusion coefficient of DNMT1 and increased its fraction bound in S-phase cells (Supplementary Movie SM15 and SM16, Supplementary Fig. S11).

In aggregate, these results demonstrate that the inhibition of DNMT1 by GSK, 5-azaC and decitabine is preceded by increased DNMT1-chromatin binding, which must be nonproductive given that the drugs are inhibitory. Yet their detailed mechanisms of inhibition are quite different, as will be discussed below.

## Discussion

In this study, we show that DNMT1 mobility is altered *in vivo* under different physiological conditions, and we explore how these perturbations of mobility relate to DNMT1 function. We find that during S phase of the cell cycle, ∼ 12% of DNMT1 molecules become less mobile and attached to chromatin, which presumably reflects their binding to the DNA replication machinery. We find it remarkable that such a modest level of DNMT1 binding is sufficient to maintain DNA methylation. Subsequently, in the G2 phase, these DNMT1 molecules are released into the nucleoplasm and become highly mobile. Pharmacological inhibition of DNMT1 by three different small molecules (GSK, 5-azaC and decitabine) dramatically limits the motion of freely diffusing DNMT1 molecules in nuclei, which is accompanied by an increase in DNMT1 molecules bound to chromatin. The chromatin binding must be nonproductive, because these drugs inhibit enzyme action. Importantly, the mechanism of GSK inhibition is very different from the covalent inhibition caused by 5-azaC and decitabine, and these drugs alter the dynamics of DNMT1 in different ways.

Our live cell single molecule imaging fills a knowledge gap concerning the well-studied DNMT1 enzyme, because it characterizes *in vivo* DNMT1 dynamics through the cell cycle and in the presence of inhibitory pharmaceutical agents. We used genome editing to express Halo-tagged DNMT1 from its endogenous loci, thereby avoiding the potential artifacts of overexpression. We tracked single DNMT1 molecules over short time frames (10 s) with a high frame rate (∼ 97 fps). This is important because ∼ 75% of DNMT1 molecules have a relatively high diffusion coefficient (> 2 µm^2^/s), and the subtleties in DNMT1 dynamics would never be captured with conventional epifluorescence imaging or single molecule imaging at lower frame rates.

Although some previous studies demonstrated that DNMT1 mobility is reduced in the S phase of the cell cycle, there are some important caveats to consider. In one study, the authors overexpressed GFP-tagged DNMT1 to study the behavior of DNMT1 in S and G2 phases of the cell cycle by live cell imaging, but their data could not capture single molecule binding events [13], because the movies were taken at a lower frame rate. Another study relied on FRAP to determine the association of DNMT1 with different components of the DNA replication machinery [47]. Whilst they were able to obtain the relative kinetics of DNMT1 association with these factors and model the *in vivo* diffusion behavior of DNMT1 molecules, there was no direct visualization of DNMT1 protein at the single molecule level. For both studies [13, 47], while they showed that DNMT1 mobility decreases in S phase (via binding to chromatin by association with DNA replication machinery), the fraction of bound DNMT1 molecules was overestimated because freely diffusing molecules were not as easily observed when imaging at lower frame rates. Consistent with this, another study imaging at lower frame rates (such as 0.0083 frames per minute) has made similar observations [52].

In our live cell single molecule imaging setup and Halo-tagged DNMT1 U2OS cell line, we show that only ∼12% DNMT1 molecules become less mobile during S phase, and the majority of DNMT1 (>70%) are still extremely mobile in the nuclei and not engaged with the replication machinery. Whether the rapidly diffusing DNMT1 provides a pool of molecules that cycles on and off the replication forks seems possible but cannot be assessed in the limited timespan of our movies.

Contrary to a previous report, our results show that DNMT1 molecules are more mobile in the G2 phase compared to asynchronous (mostly G1) cells. Previous work had suggested that DNMT1 is more chromatin-bound during G2 [13]. However, live cell imaging of overexpressed GFP-tagged DNMT1 protein domains may have complicated the interpretation of those results. Our results show that although ∼12% of DNMT1 is bound to chromatin/DNA replication machinery at any one time in S phase, these molecules are then released into the nucleoplasm in G2 and become more mobile before re-associating with chromosomes at the metaphase plate for cell division.

Disordered regions rich in acidic amino acids are found on many nuclear proteins. Here, we mutated the acidic amino acids on the *N*-terminus of DNMT1, including its IDR, to test for effects on protein mobility and chromatin binding. Unexpectedly, mutations in both the IDR region (D/E-100-400) and α-helical domain (D/E-22-91) that neutralize the negative charges lead to dramatic relocalization of DNMT1 to chromatin. In agreement with our results, one study suggested that the Asp/Glu-rich IDR in HMGB1 prevents its association on non-cognate sites, therefore accelerating its search for cognate targets [53]. The authors suggest that this model may extend to other DNA-binding proteins that contain such acidic IDRs, as now demonstrated by DNMT1.

There are two possibilities that may explain the dynamics of our DNMT1 *N*-terminal mutants. The IDR of DNMT1 may facilitate the dissociation of DNMT1 from chromatin either directly through its negative charge or indirectly by promoting association with other nucleoplasmic proteins. The DMAP1 transcriptional repressor [21, 22] may be one such protein. The D/E mutations would then be disrupting these features that promote release of DNMT1 from chromatin. This may be because this non-canonical IDR is enriched in glutamic and aspartic acids, rather than the usual serine, proline and tyrosine found in other phase-separating proteins. Alternatively, these D/E-mutations may have a gain-of-function phenotype, promoting DNMT1 association with other chromatin proteins. Based on our results, where mutations of both the α-helical domain and the IDR have an additive effect on the mobility of DNMT1 (compare the D/E-22-400 mutant versus D/E-22-91 or D/E-100-400 respectively), we suspect that a combination of the IDR and binding of other proteins normally facilitates DNMT1 relocalization from chromatin to the nucleoplasm. Future work determining what proteins are bound to various region of the *N*-terminal domain at different cell cycle phases will be required to understand how the domain contributes to DNMT1 dynamics and function.

We also show that acute treatment of cells with small molecule inhibitors dramatically alters DNMT1 dynamics in the nucleus, even at early timepoints (4 h) when DNA methylation levels are minimally affected. Despite having different inhibitory mechanisms, fundamentally both GSK and 5-azaC decrease DNMT1 mobility and significantly increase the fraction bound to chromatin from ∼ 12% to ∼ 20%. This increase in chromatin binding following drug treatment was expected for 5-azaC (as it is a nucleoside analog that acts as a suicide inhibitor), especially after longer times when it has been incorporated into DNA. However, it was unexpected for the GSK drug. These results imply that small molecule inhibition of DNMT1 causes non-productive binding to chromatin. In agreement with these ideas, one study showed that following treatment with the decitabine, DNMT1 co-purified with DNA adducts in a cesium chloride gradient [54]. An open question is whether and how increased chromatin binding of DNMT1 (at 4 h post small inhibitor treatment) leads to proteasomal degradation and decreased DNA methylation (at > 24 h treatment). Interestingly, using both live cell single molecule imaging and biochemical approaches, two other groups showed that small molecule inhibition of other nuclear proteins causes similar relocalization of previously nucleoplasmic protein to chromatin [49, 55]. This trend is observed despite these proteins having very different functions in the cell and their specific small molecule inhibitor using very different mechanisms of inhibition. Future work will be required to elucidate if some common mechanism applies to these diverse examples.

Our work shows a cell cycle dependence for DNMT1 dynamics by the nucleoside analogs 5-azaC and decitabine, consistent with these drugs becoming incorporated into DNA during DNA replication in the S phase of the cell cycle. As 5-azaC is converted to 5-aza-deoxyC in cells, these observations have implications for treatment protocols for decitabine, which is currently used in the treatment of myelodysplastic syndromes and myeloid leukemia [24–28]. To the extent that these inhibitors function by inducing DNA hypomethylation, they should be effective when cells are in S phase and may have reduced efficacy if used in combination with another treatment that blocks DNA replication.

Importantly, one study showed that the formation of covalently linked decitabine-DNA adducts perturbs mitosis independent of DNA demethylation and that this may be a key mechanism of inhibition [56]. Decitabine is linked to loss of sister chromatid cohesion during metaphase and increased cell aneuploidy. Moreover, decitabine-DNA adducts activate the DNA damage response during the intra-S phase checkpoint [56]. Consistent with these ideas, although decitabine binds to DNMT1 and causes hypomethylation in AML patients, only a fraction of patients is responsive to decitabine treatment and these responses often tend to be short lived, especially when chemotherapeutic treatment is stopped [57].

For ease of imaging, human osteosarcoma U2OS cells were used in this study. Although DNMT1 is an essential housekeeping enzyme, its protein levels and activity differ among cell types [58–64], so whether the dynamics seen here for U2OS cells would apply to other cell types remains to be determined.

In aggregate, our results demonstrate the *in vivo* dynamics of DNMT1 under a variety of conditions both physiological and pharmacological. Specifically, this live cell single molecule imaging setup is able to capture the movement of fast diffusing DNMT1 molecules *in vivo* (> 2 µm^2^/s), which was not previously possible with conventional epifluorescence imaging techniques. In general, decreased DNMT1 mobility in the S phase of the cell cycle is accompanied by DNMT1 binding to chromatin/DNA, which has implications for the DNA methyltransferase activity of DNMT1 and DNMT1 functioning in transcriptional regulation.

## Supplementary Material

gkag089_Supplemental_Files

## Data Availability

Nanopore DNA sequencing data underlying this article can be accessed from the GEO repository (GSE299486). Uncropped blots and gels are presented in Supplementary Fig. S12. Other data underlying this article will be shared on reasonable request to the corresponding author.

## Notes

Present address: University of California San Diego, La Jolla, CA, 92093, USA
