# Supplementary material for: Single-molecule tracking of DNMT1 in living cells reveals its cell cycle dynamics and its redistribution upon drug treatment
Source: Nucleic Acids Res. 2026 Feb 5;54(4):gkag089. doi: 10.1093/nar/gkag089 (PMC12873607; doi:10.1093/nar/gkag089)
Supplement: gkag089_Supplemental_Files [file gkag089_supplemental_files.zip › Lee_Supplementary_data_resubmit3.pdf]

Supplementary Data

Single-molecule tracking of DNMT1 in living cells reveals its cell cycle dynamics and its redistribution upon drug treatment

Eliza S. Lee[1,2,3], Ella R. Tommer[1,2], Paul B. Rothman[1,2,4], Sarah V. Middleton[2,5], Daniel T. Youmans [1,2,4,6], Thomas R. Cech[1,2,3]

1 - BioFrontiers Institute, University of Colorado Boulder, Boulder, CO 80303, USA

2 - Department of Biochemistry, University of Colorado Boulder, Boulder, CO 80303 USA

3 - Howard Hughes Medical Institute, University of Colorado Boulder, Boulder, CO 80303, USA

4 - John Hopkins School of Medicine, Baltimore, MD 21205, USA

5 - Department of Molecular, Cellular and Developmental Biology, University of Colorado Boulder, Boulder, CO 80303, USA

6 - Present address: University of California San Diego, La Jolla, CA, 92093, USA

Corresponding email: thomas.cech@colorado.edu

This file contains:

12 Supplementary Figures (S1 to S12) with figure legends

Supplementary Methods

4 Supplementary Tables (Supplementary Table 1 to 4)

16 Supplementary Movies (labelled SM1 to SM16)

Supplementary Figures

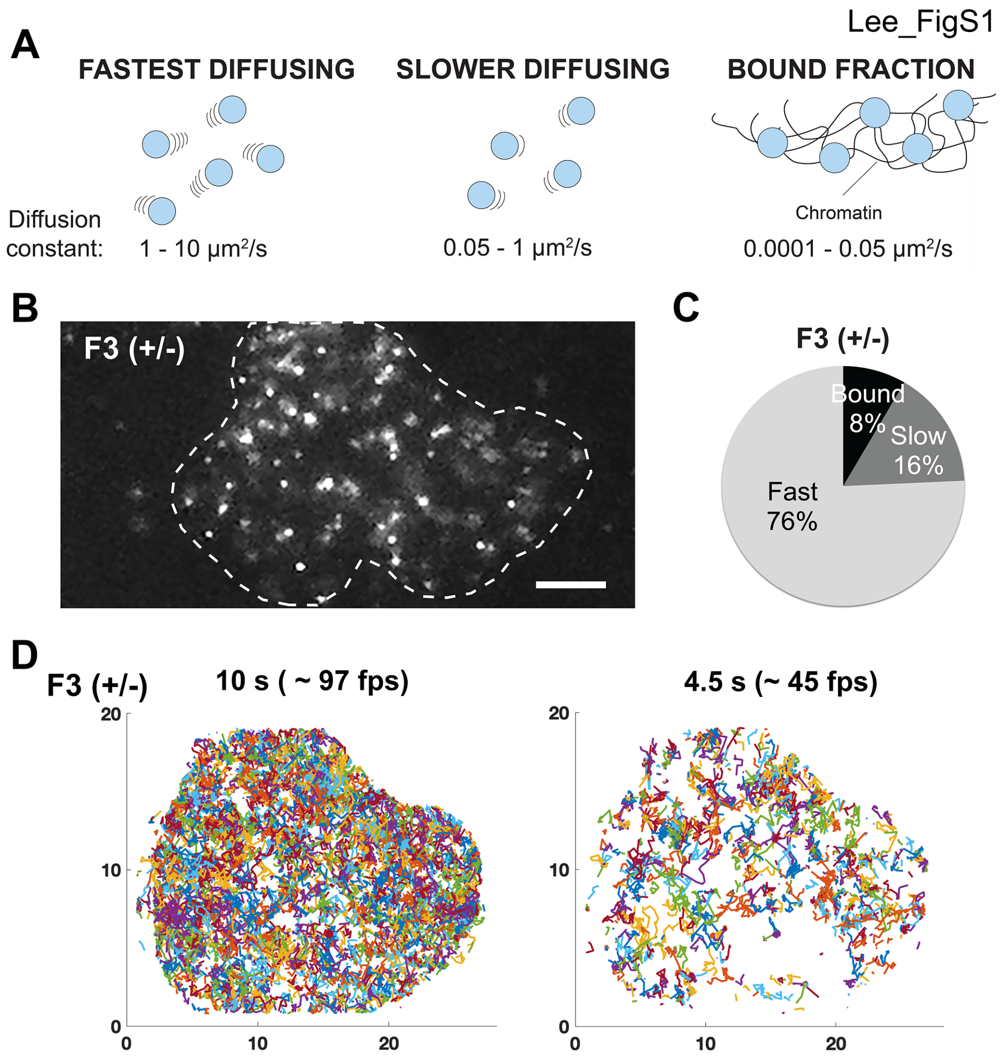

Supplementary Figure S1. Spot-on model and trajectories of DNMT1 in live cell single molecule imaging **(related to Fig 2)**.

(A) Three state model used in this study. (B) Still from representative Halo-tagged DNMT1 F3 (+/-) movie (composite of 5 frames, ~ 0.5 s) showing the DNMT1 molecules in each nucleus, similar to Fig 2A. Dotted line denotes outline of the nucleus, scale bar = 5 μm.

(C) Pie chart showing the distribution of fast or slow diffusing and chromatin-bound DNMT1 molecules in asynchronous F3 (+/-) cells, similar to Fig 2F.

(D) Representative nuclei showing the trajectories of DNMT1 molecules in asynchronous F3 (+/-) cells. Left, all trajectories shown in a 10 s movie taken. Right, a subset of the trajectories from the last 4.5 s of the corresponding movie.

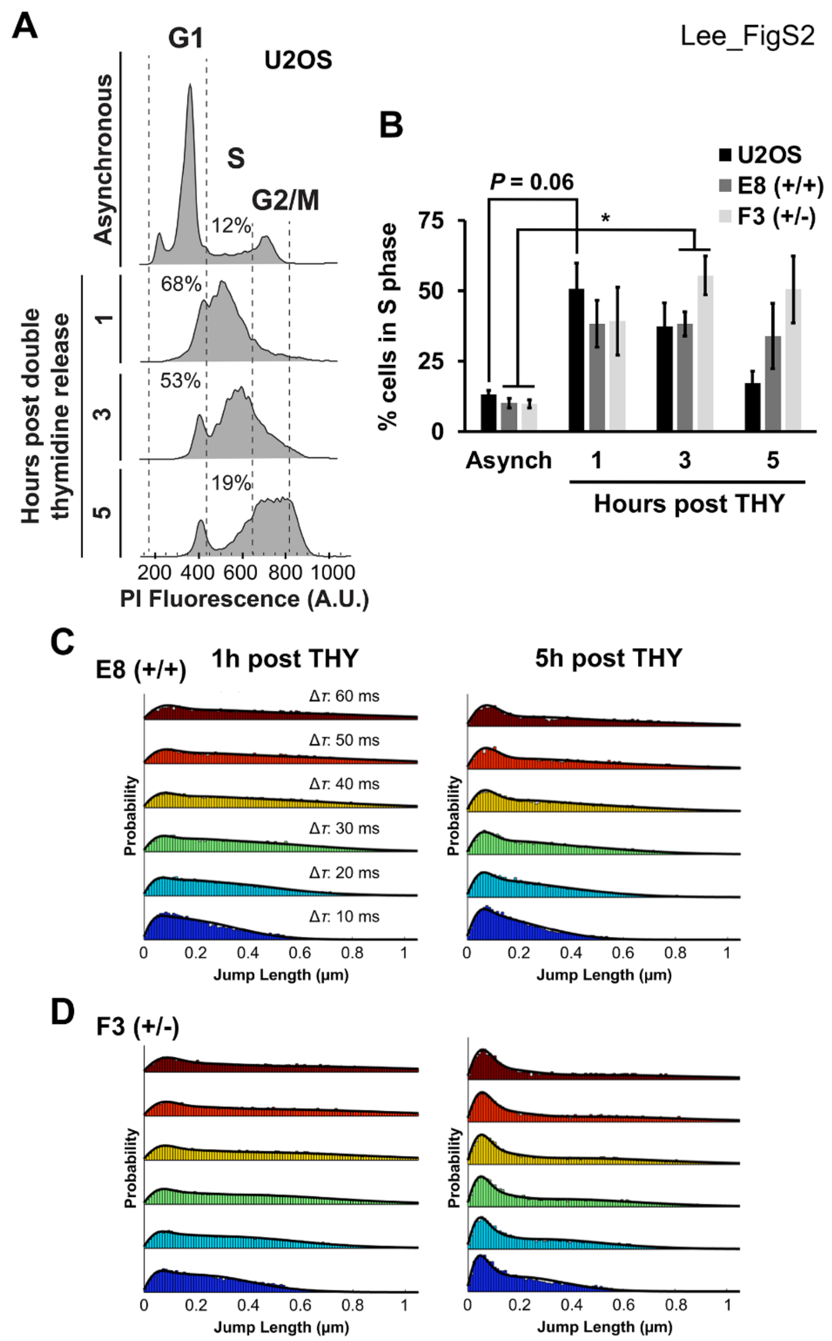

Supplementary Figure S2. Cell cycle synchronization and Spot-on analysis of DNMT1 dynamics in S phase. **(related to Figure 3)**

(A) Flow cytometry of propidium iodide stained cells to show cell cycle progression (based on the DNA content of the cells). Numbers give percent of cells in S phase. Following thymidine release, there is an increase in S phase cells which culminates at 1-3 h post release, related to Fig 3B.

(B) The percentage of S phase cells is shown at various time points post thymidine release is shown here. Student t-test performed, * $P < 0.05$.

(C-D) Displacement histograms of DNMT1-Halo molecules in S phase (related to Fig 3C-I). Representative displacement histograms for multiple $\Delta\tau$ (residence time) of DNMT1-Halo tagged molecules in E8 (+/+) and F3 (+/-) cells synchronized to either early S phase (1 h post thymidine) or mid-S (5 h post thymidine).

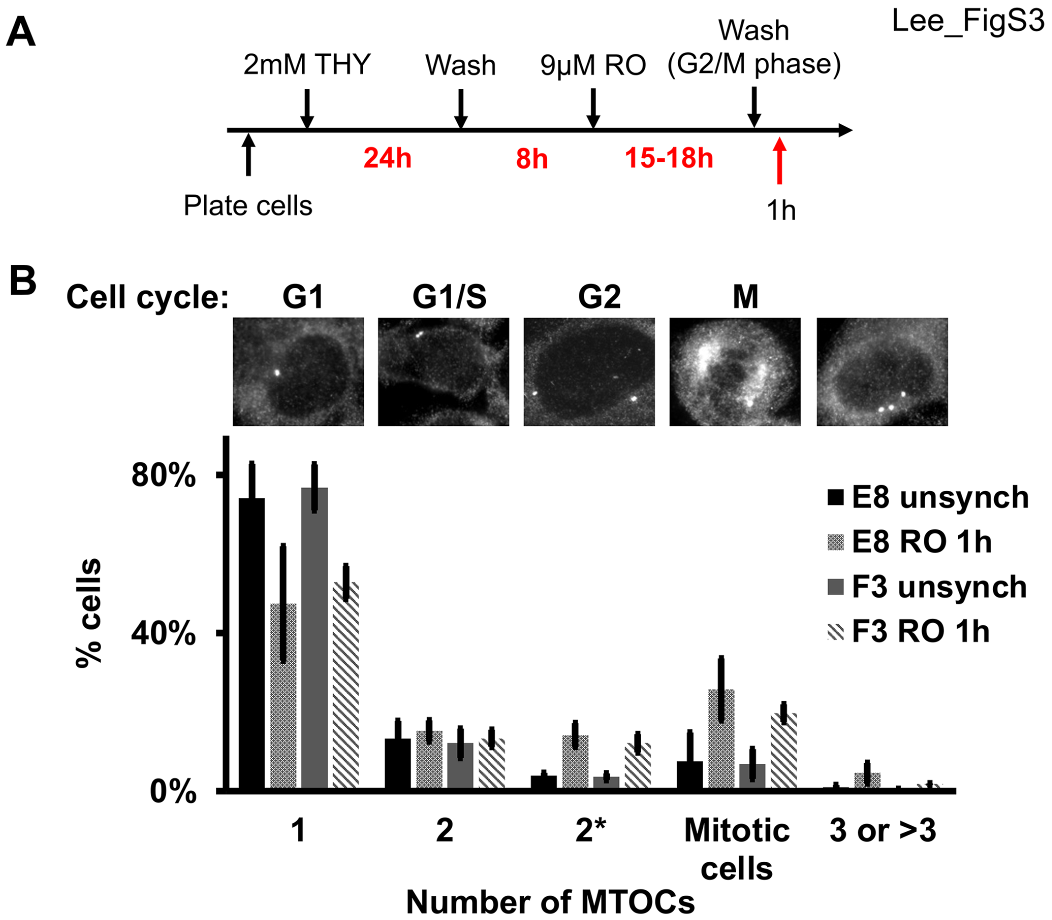

Supplementary Figure S3. Synchronizing U2OS cells to G2/M phase of the cell cycle

(A) RO-3306 protocol used to synchronize cells to G2/M phase

(B) Immunofluorescence staining of the microtubule organizing center (MTOC) protein γ-tubulin to determine cell cycle status. Cells with 2 MTOCs on opposite sides of the nucleus (denoted as 2*) are likely to be in G2 phase of the cell cycle, whereas cells with only one MTOC are in G1 phase of the cell cycle. Cells with 2 MTOCs next to each other are in G1/S phase. 200-400 cells were scored for each cell line and across at least four biological replicates. Vertical bars represent the percentage of cells with varying MTOCs number and error bars represent standard error of mean of different replicates.

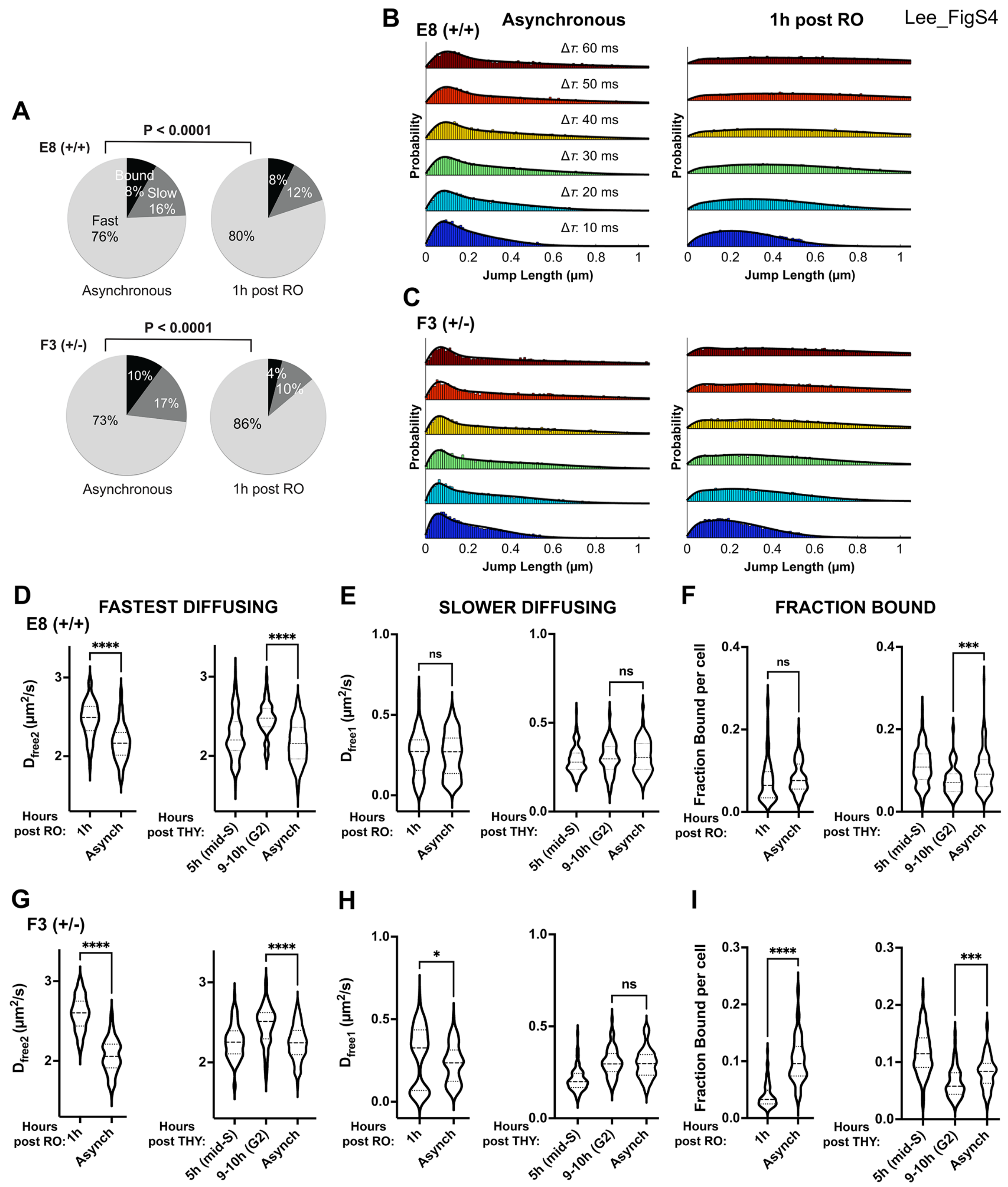

Supplementary Figure S4. DNMT1 dynamics in the G2 phase of the cell cycle.

Cells were synchronized using RO-3306 to G2/M phase of the cell cycle. (A) Pie charts showing the distribution of fast or slow diffusing and chromatin-bound DNMT1 molecules in asynchronous cells or 1 h post RO release. (B-C) Representative displacement histograms of DNMT1-Halo tagged molecules in E8 (+/+) and F3 (+/-) cells that are either asynchronous or synchronized to G2/M (1 h post RO release). (D-I) Violin plots showing the distribution of DNMT1-Halo in 86-93 cells across 3 replicates. (D-E) and (G-H) G2 cells (1h post RO release)

have consistently faster DNMT1-Halo molecules than asynchronous cells for both the fastest and slower diffusing populations. (F, I) Fraction of DNMT1-Halo that is chromatin-bound in G2/M (compare left two violin plots) and S phase (compare right three violin plots). Student t-test, two-tailed, **** $P < 0.0001$, *** $P < 0.001$, * $P < 0.05$, ns = not significant.

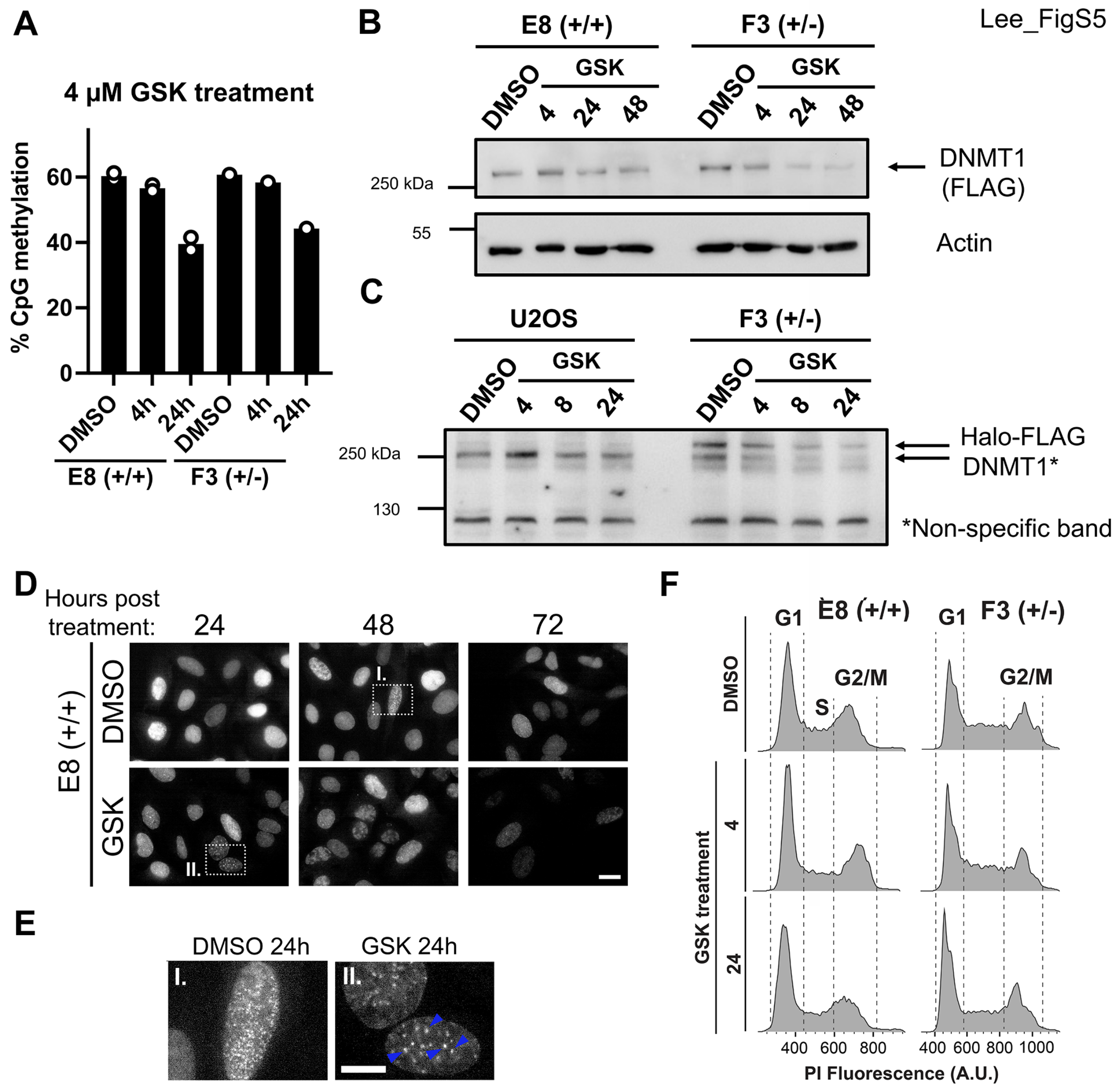

Supplementary Figure S5. Treatment of cells with GSK decreases DNA methylation levels, causes DNMT1 degradation, and leads to punctate DNMT1 foci in a subset of the treated cells, but neither GSK nor 5-azaC inhibits the activity of purified DNMT1 *in vitro* **(related to Figure 5)**.

(A) % CpG methylation of gDNA extracted from cells treated with DNMT1-specific inhibitor GSK for 4 h, 24 h or DMSO. Related to Fig 5A.

(B) Immunoblot similar to Fig 5B, but probed with anti-FLAG antibody, showing that DNMT1 levels decreases following GSK treatment in either E8 (+/+) or F3 (+/-) cells. Actin is used as a loading control.

(C) Immunoblot probed with anti-DNMT1 antibody shows that endogenous DNMT1 levels decreases following GSK treatment in both the parental U2OS and F3 (+/-) cells. Unspecific band (~ 120 kDa) is used as a loading control.

(D, E) The subcellular localization of Halo-tagged DNMT1 is not altered following GSK treatment. E8 (+/+) or F3 (+/-) cells were treated with either DMSO or GSK for the indicated time points, and DNMT1 was visualized by incubating cells with 500 μM JF546 dye for 5 min before fixation. Representative images, Scale bar = 20 μm. (E) Higher magnification image of cells indicated in (D), Scale bar = 10 μm. Punctate DNMT1-foci (denoted by blue arrows) are observed following GSK drug treatment.

(F) Flow cytometry of propidium iodide-stained cells to show cell cycle progression (based on the amount of DNA present in the cells). The cell cycle progression does not change substantially following GSK drug treatment.

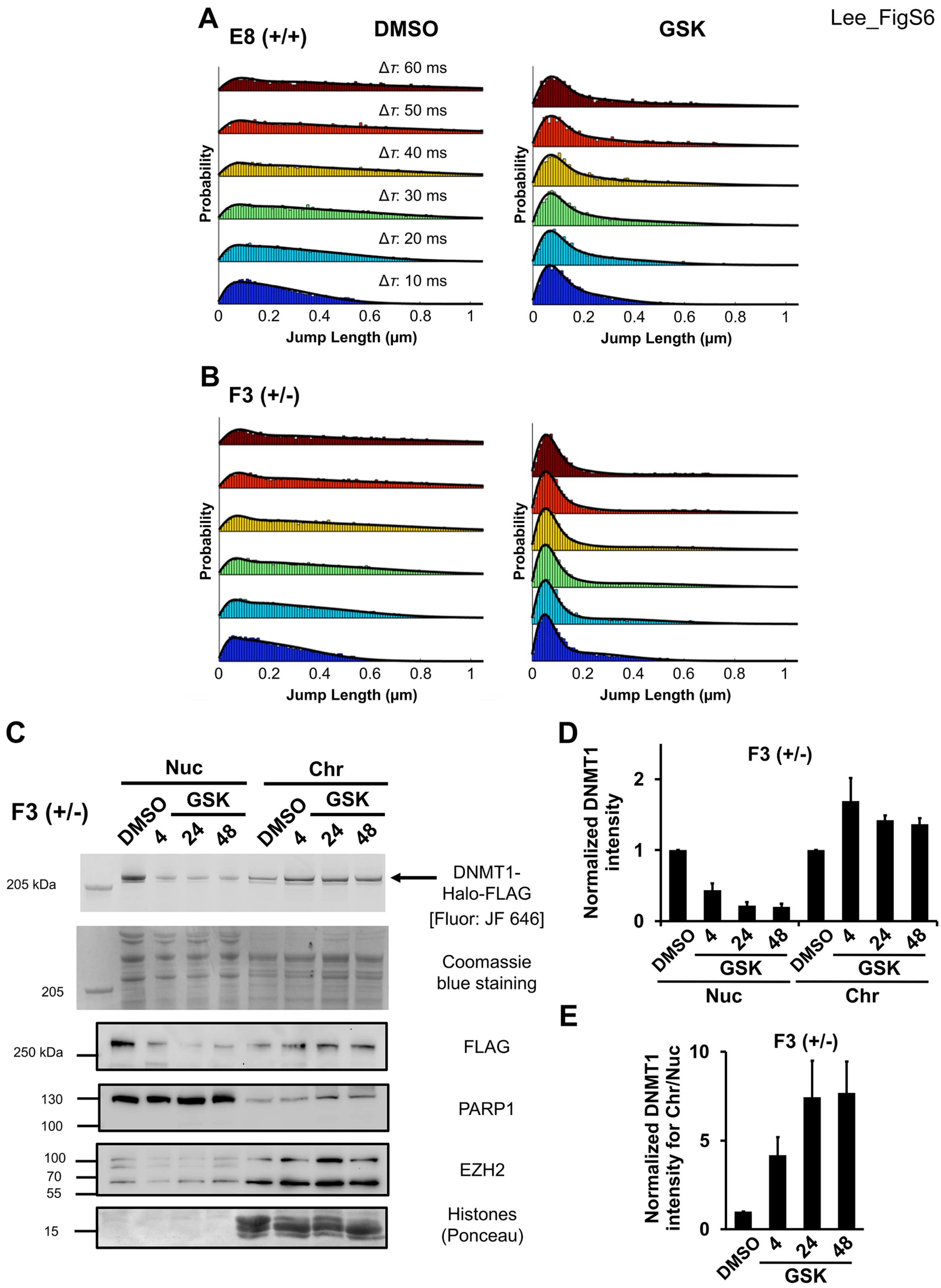

Supplementary Figure S6. Acute treatment of cells with GSK significantly decreases DNMT1 mobility and redistributes it from nucleoplasm to chromatin **(related to Figure 5)**.

(A-B) Representative displacement histograms of DNMT1-Halo tagged molecules in (A) E8 (+/+) or (B) F3 (+/-) cell lines DMSO- or GSK-treated for 4 h.

(C-E) GSK treatment relocalizes DNMT1 from the nucleoplasm to chromatin, similar to Figure 5G-I. (C) F3 (+/-) cells either DMSO- or GSK-treated were fractionated into nucleoplasm and chromatin fractions and DNMT1-Halo intensity was visualized by a JF646 fluorescent gel or immunoblot against FLAG. Note that the steady state levels of DNMT1 in the nucleoplasm decrease following GSK treatment, consistent with total steady state levels shown in Figure 5B. PARP1 and histones (from Ponceau staining) are used protein markers for each fraction. (D) Relative DNMT1 intensity, normalized to total protein loading, from JF646 fluorescent gel, error bars represent standard error of mean for 4 replicates, see Supplementary Fig S7. (E) DNMT1 intensity in the chromatin fraction divided by that in the nucleoplasmic fraction.

Lee_FigS7

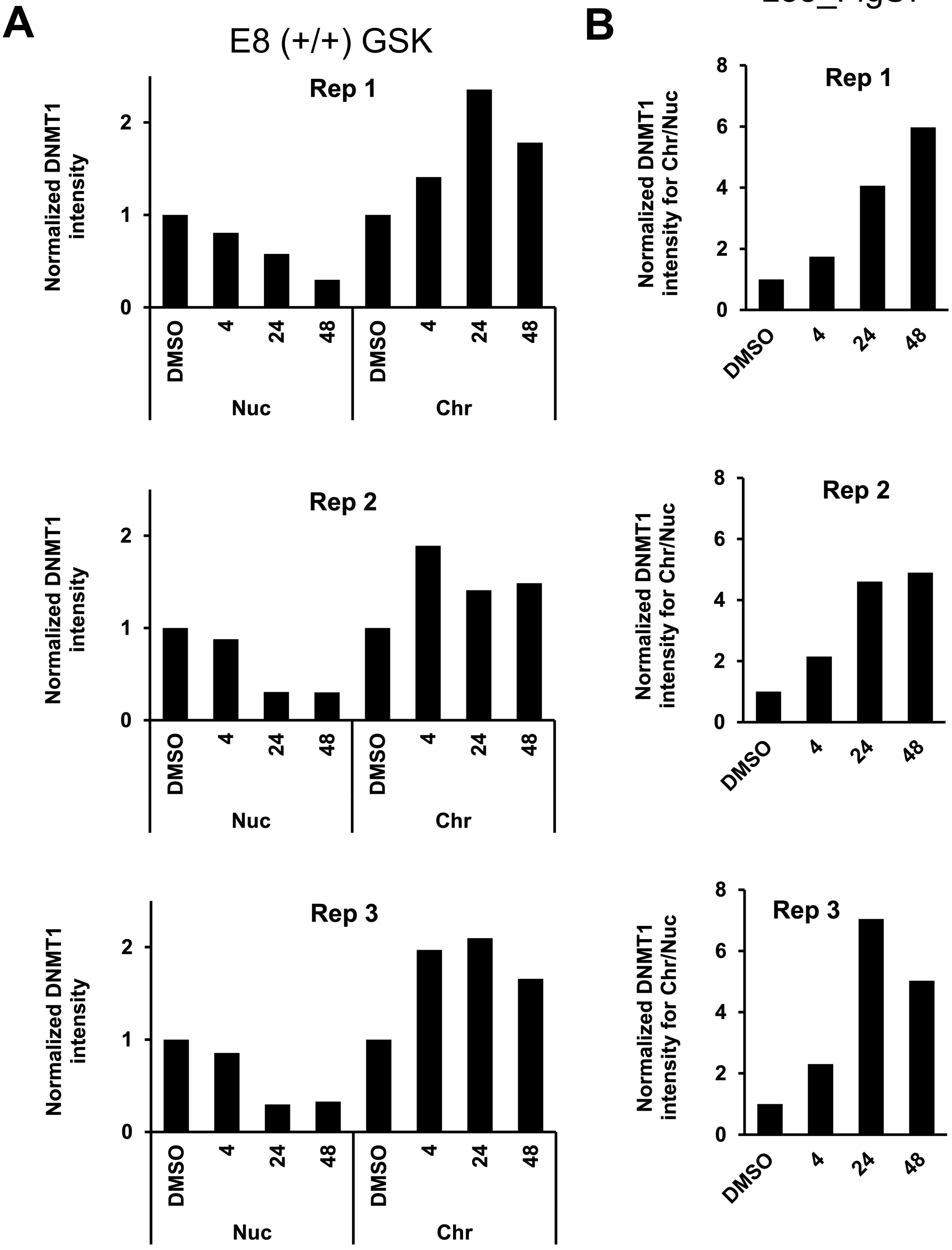
A
E8 (+/+) GSK
Rep 1
Rep 2
Rep 3
Normalized DNMT1 intensity
0
1
2
DMSO
4
24
48
Nuc
Chr
B
Rep 1
Rep 2
Rep 3
Normalized DNMT1 intensity for Chr/Nuc
0
2
4
6
8
DMSO
4
24
48

Lee_FigS7

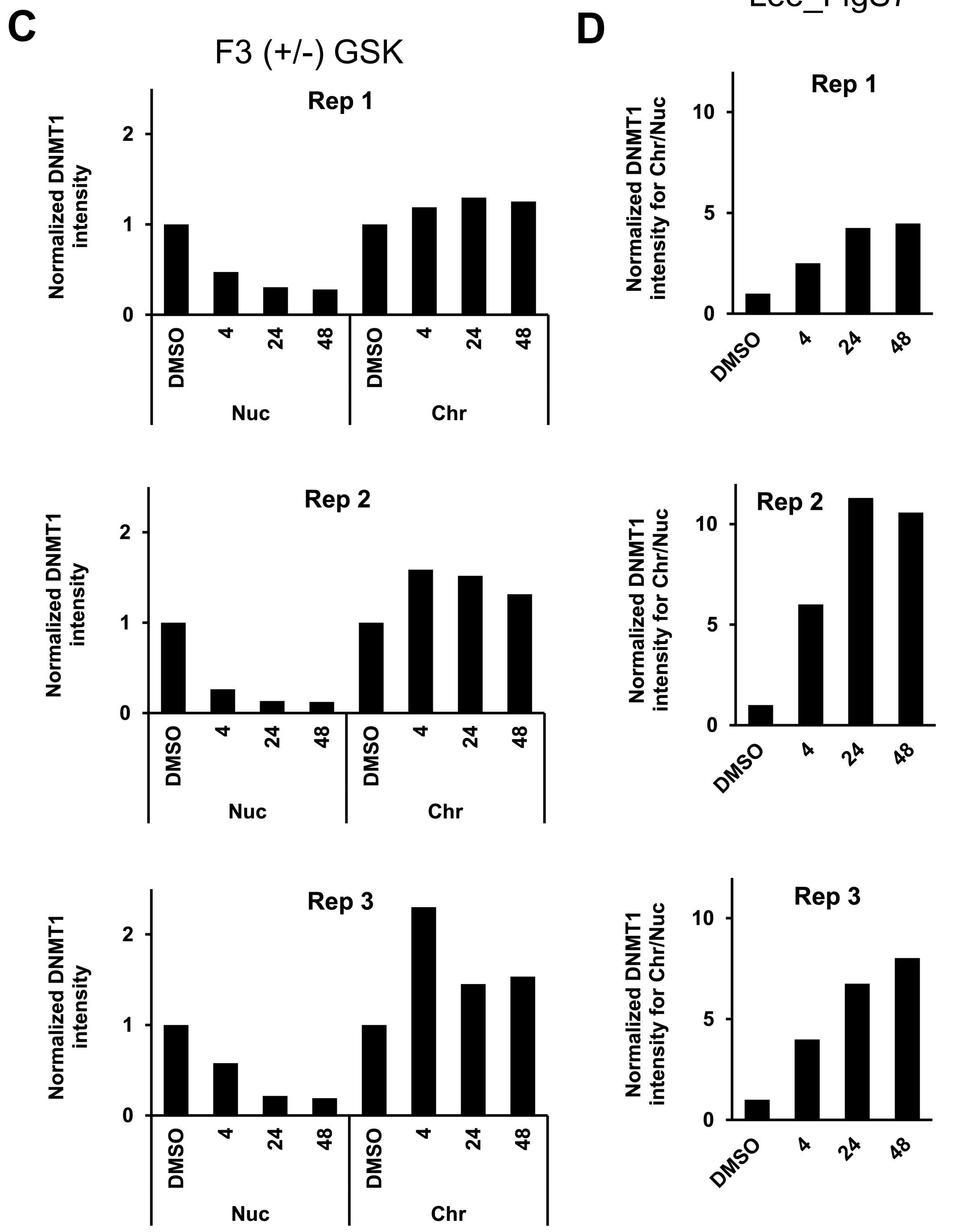

Supplementary Figure S7. Replicates for quantifying DNMT1 content in nucleoplasm and chromatin fractions. DNMT1 intensity (normalized to total protein loading) in (A, B) GSK-treated E8 (+/+) cells (related to Figure 5G-I); (C-D) GSK-treated F3 (+/-) cells (related to Supplementary Figure S6C-E).

Lee_FigS8

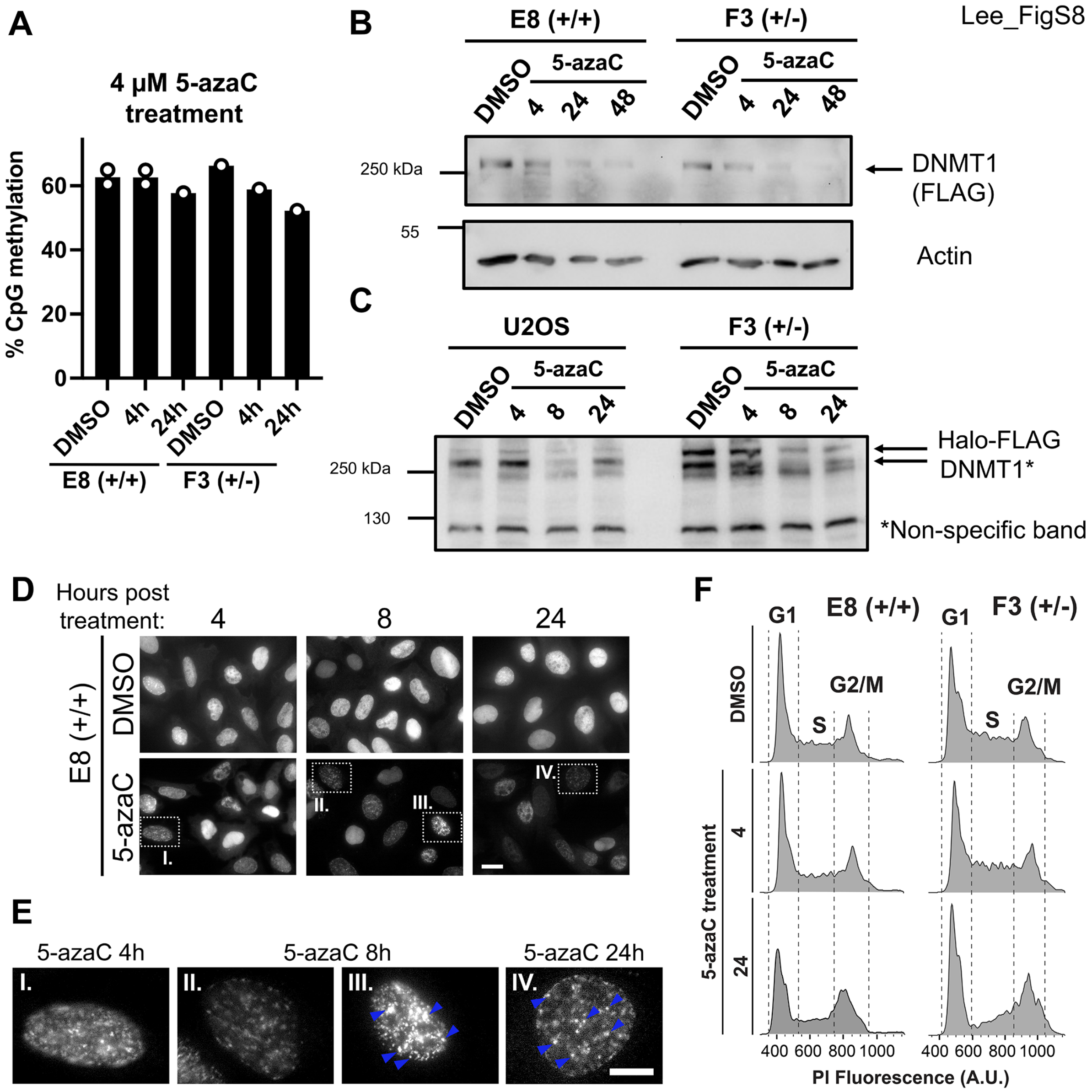

Supplementary Figure S8. Treatment of cells with 5-azaC decreases DNA methylation levels, causes DNMT1 degradation, and leads to punctate DNMT1 foci in a subset of the treated cells **(related to Fig 6)**.

(A) CpG methylation levels in gDNA extracted from cells treated with DNMT1 inhibitor 5-azaC for 4 h, 24 h or with DMSO. Related to Fig 6A.

(B) Immunoblot for FLAG-tagged DNMT1 similar to Fig 6B, showing that DNMT1 levels decreases following 5-azaC treatment in either E8 (+/+) or F3 (+/-) cells. Actin is used as a loading control.

(C) Immunoblot using DNMT1 antibody shows that endogenous DNMT1 decreases following 5-azaC treatment in either the parental U2OS or F3 (+/-) cells. Unspecific band (~ 120 kDa) is used as a loading control.

(D, E) The subcellular localization of Halo-tagged DNMT1 does not change following 5-azaC treatment. Cells were treated with either DMSO or 5-azaC for the indicated time points, and DNMT1 was visualized by incubating cells with 500 nM JF546 dye for 5 min before fixation. (D) Representative images, scale bar = 20 μM. (E) Higher magnification images of cells indicated in (D), scale bar = 10 μM. Punctate DNMT1 foci are denoted by blue arrows.

(F) Flow cytometry of propidium iodide-stained cells to show cell cycle progression (based on the amount of DNA present in the cells).

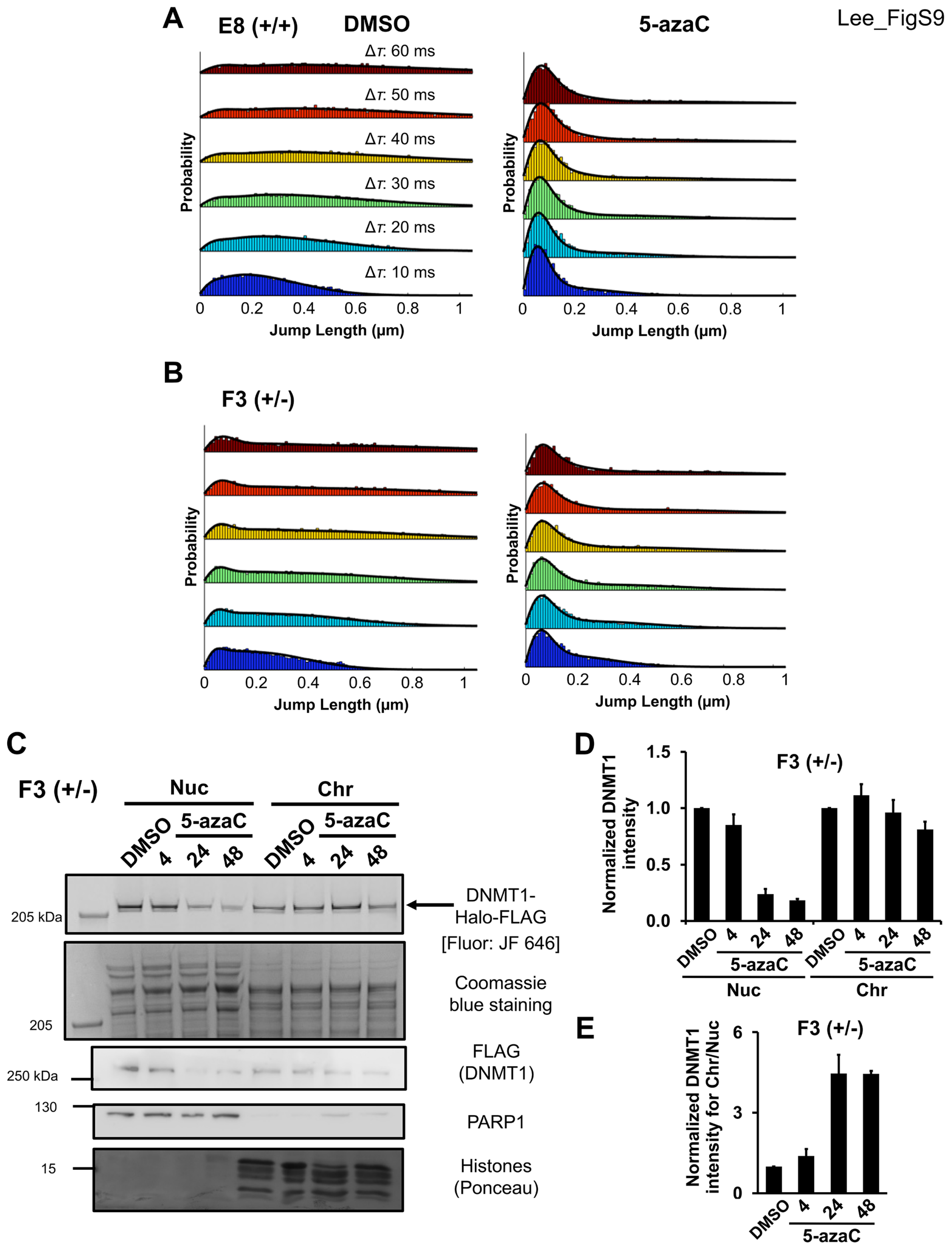

Supplementary Figure S9. Acute treatment of 5-azaC decreases the mobility of a subset of DNMT1 and causes it to redistribute from nucleoplasm to chromatin **(related to Fig 6)**.

(A, B) Representative displacement histograms of multiple Δτ (residence time) of DNMT1-Halo tagged molecules in E8 (+/+) and F3 (+/-) cell lines after DMSO or 5-azaC treatment for 4 h.

(C-E) 5-azaC treatment relocalizes DNMT1 from the nucleoplasm to chromatin, similar to Figure 6G-I. (C) F3 (+/-) cells either DMSO- or 5-azaC-treated were fractionated into nucleoplasm and chromatin fractions and DNMT1-Halo intensity was imaged by a JF646 fluorescent gel (top panel) or immunoblot against FLAG (middle panel). Note that the levels of DNMT1 in the nucleoplasm decreases following 5-azaC treatment, consistent with total steady state levels shown in Fig 6B. PARP1 and histones (from Ponceau staining) are used protein markers for each fraction. (D) Relative DNMT1 intensity, normalized to total protein loading, from the JF646 fluorescent gel, error bars represent standard error of the mean from three replicates, see Supplementary Fig S10. (E) is DNMT1 intensity in the chromatin fraction divided by the nucleoplasmic fraction.

Lee_FigS10

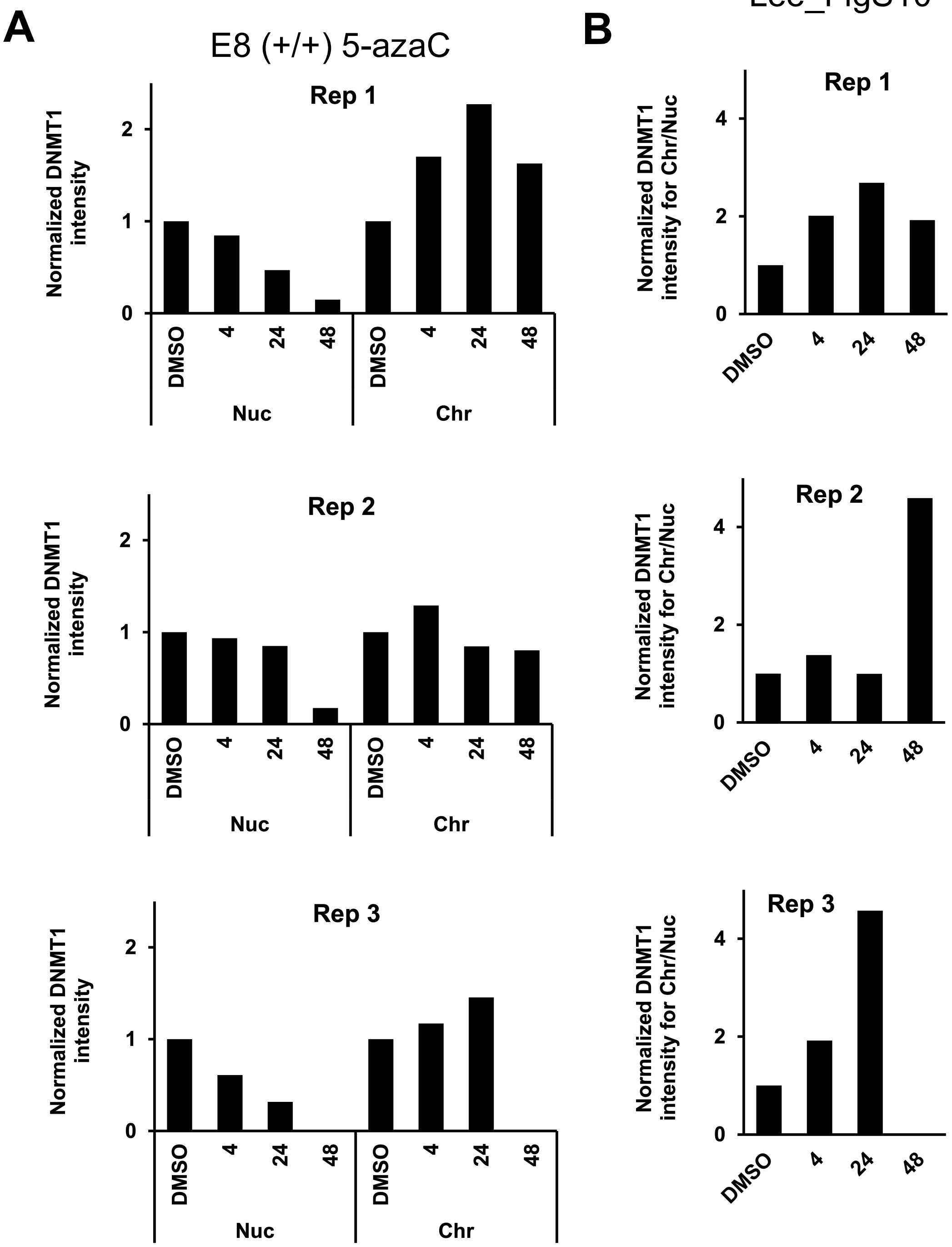
A
E8 (+/+) 5-azaC
Rep 1
Rep 2
Rep 3
Normalized DNMT1 intensity
0
1
2
DMSO
4
24
48
Nuc
Chr
B
Rep 1
Rep 2
Rep 3
Normalized DNMT1 intensity for Chr/Nuc
0
2
4
DMSO
4
24
48

Lee_FigS10

C

F3 (+/-) 5-azaC

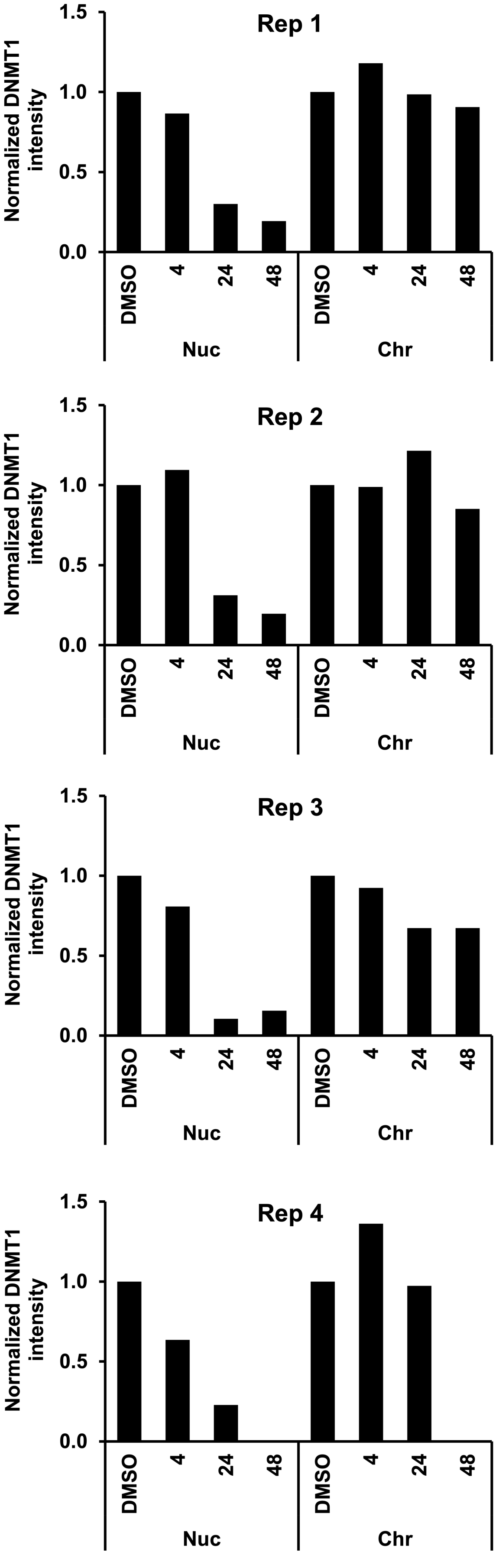

D

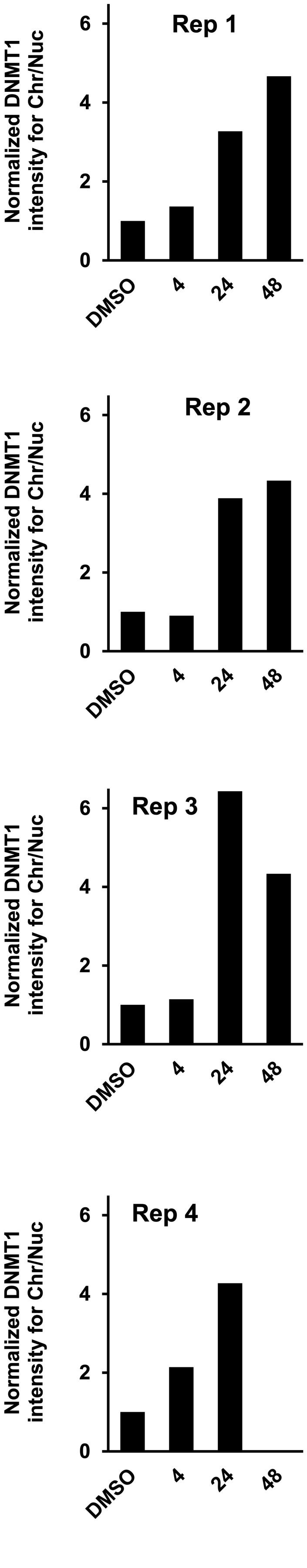

Supplementary Figure S10. Replicates for quantifying DNMT1 content in nucleoplasm and chromatin fractions. DNMT1 in intensity (normalized to total protein loading) in (A-B) 5-azaC treated E8 (+/+) cells (related to Figure 6G-I) and (C-D) 5-azaC treated F3 (+/-) cells (related to Supplementary Figure S9C-E).

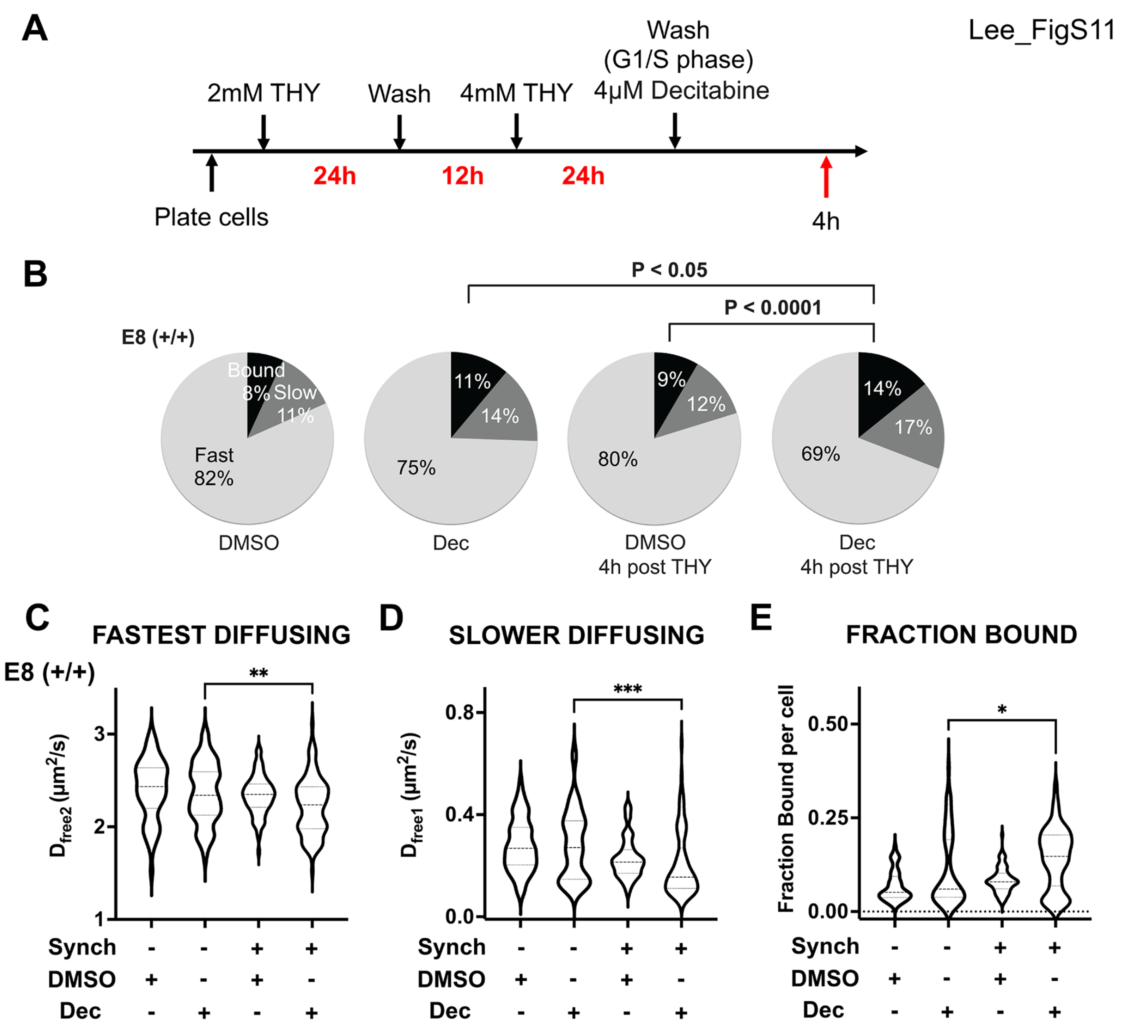

Supplementary Figure S11. Decitabine (5-aza-dC) inhibition of DNMT1 dynamics occurs in the S phase of the cell cycle **(similar to Figure 7).**

(A) Schematic of double thymidine block protocol used to synchronize cells, followed by either DMSO or 4 μM decitabine treatment for 4h.

(B-E) Spot-on analysis of cells synchronized to S phase and treated with decitabine. (B) Pie charts showing the distribution of DNMT1 molecules following DMSO and decitabine treatment in either asynchronous or synchronized (4 h post-THY) cells. (C-E) Violin plots showing the diffusion coefficients and fraction bound of DNMT1-Halo in 61-103 cells across 4 replicates. Student t-test, two-tailed, *** $P < 0.001$, ** $P < 0.01$, * $P < 0.05$.

Lee_FigS12

Fig 1B

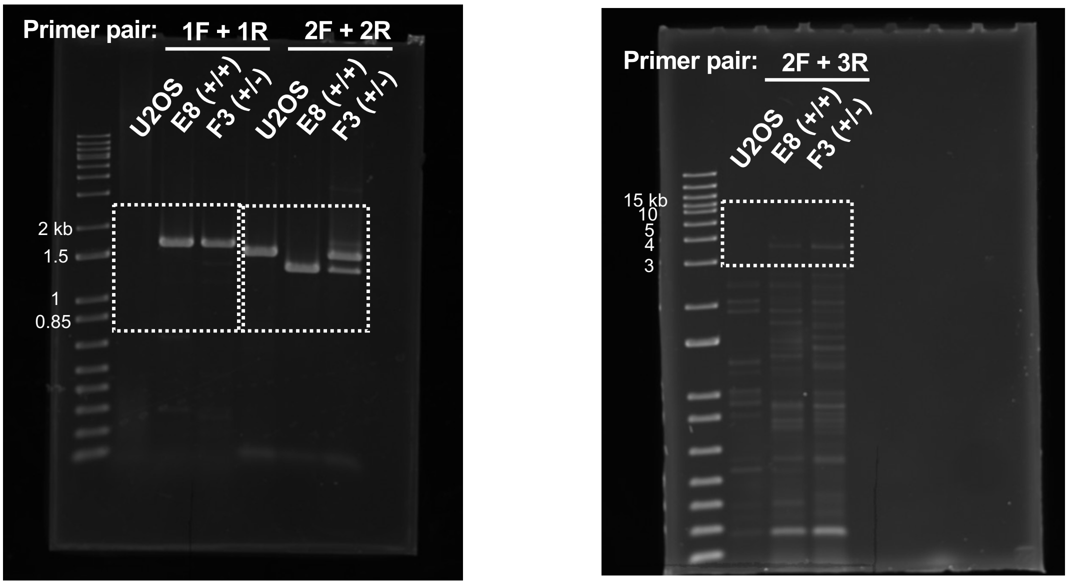

Fig 1C

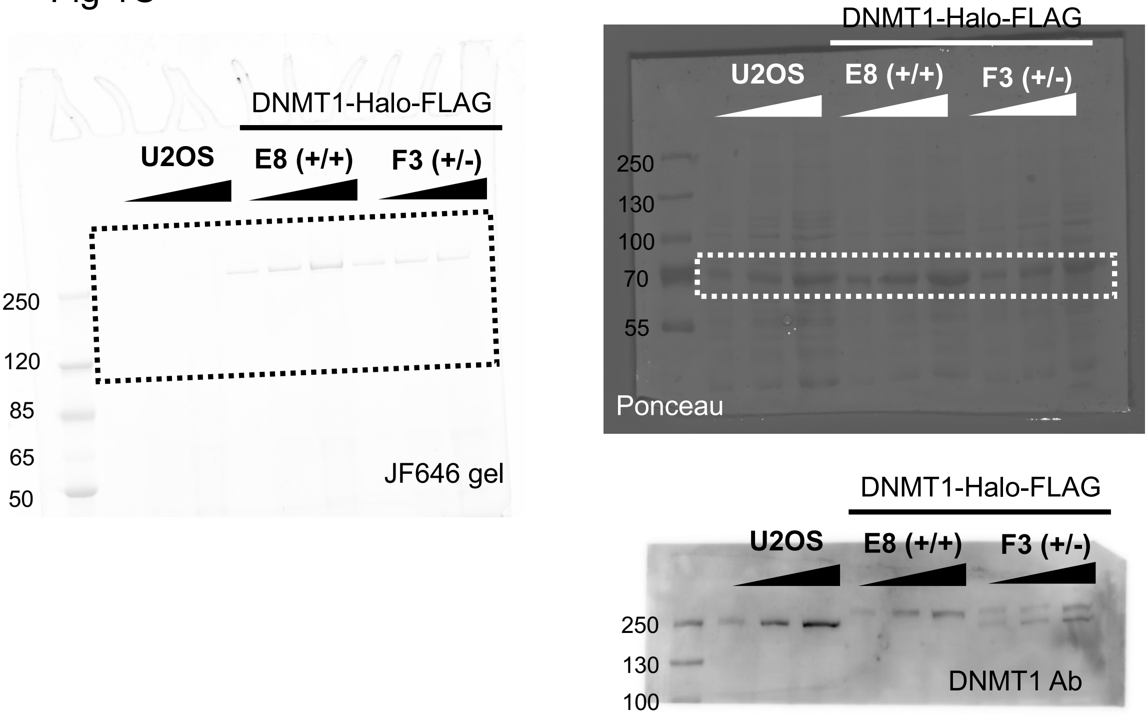

Supplementary Figure S12. Uncropped versions of gels and blots shown in this paper.

Lee_FigS12

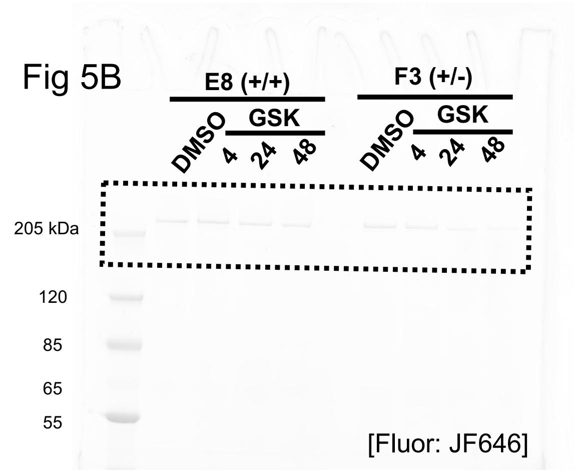

Coomassie

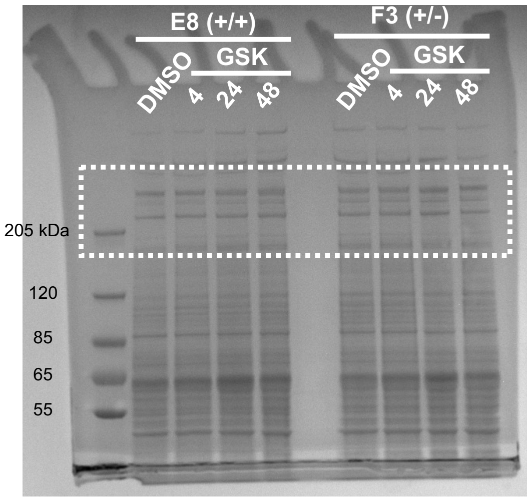

Supplementary Fig S5B

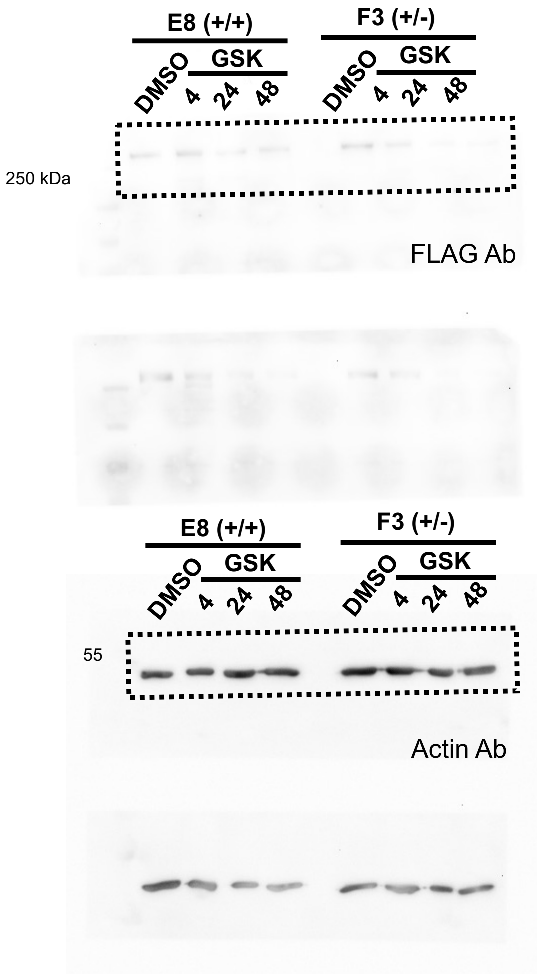

Supplementary Fig S5C

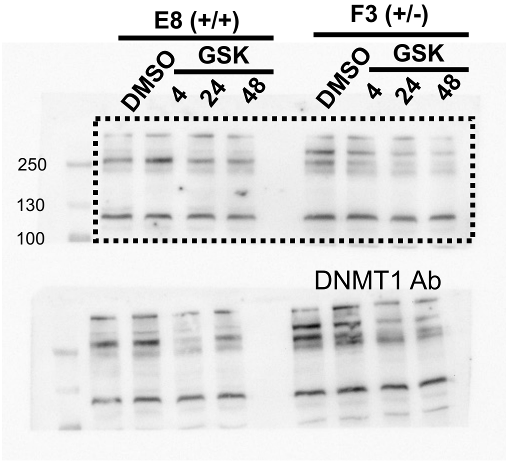

Lee_FigS12

Fig 5G

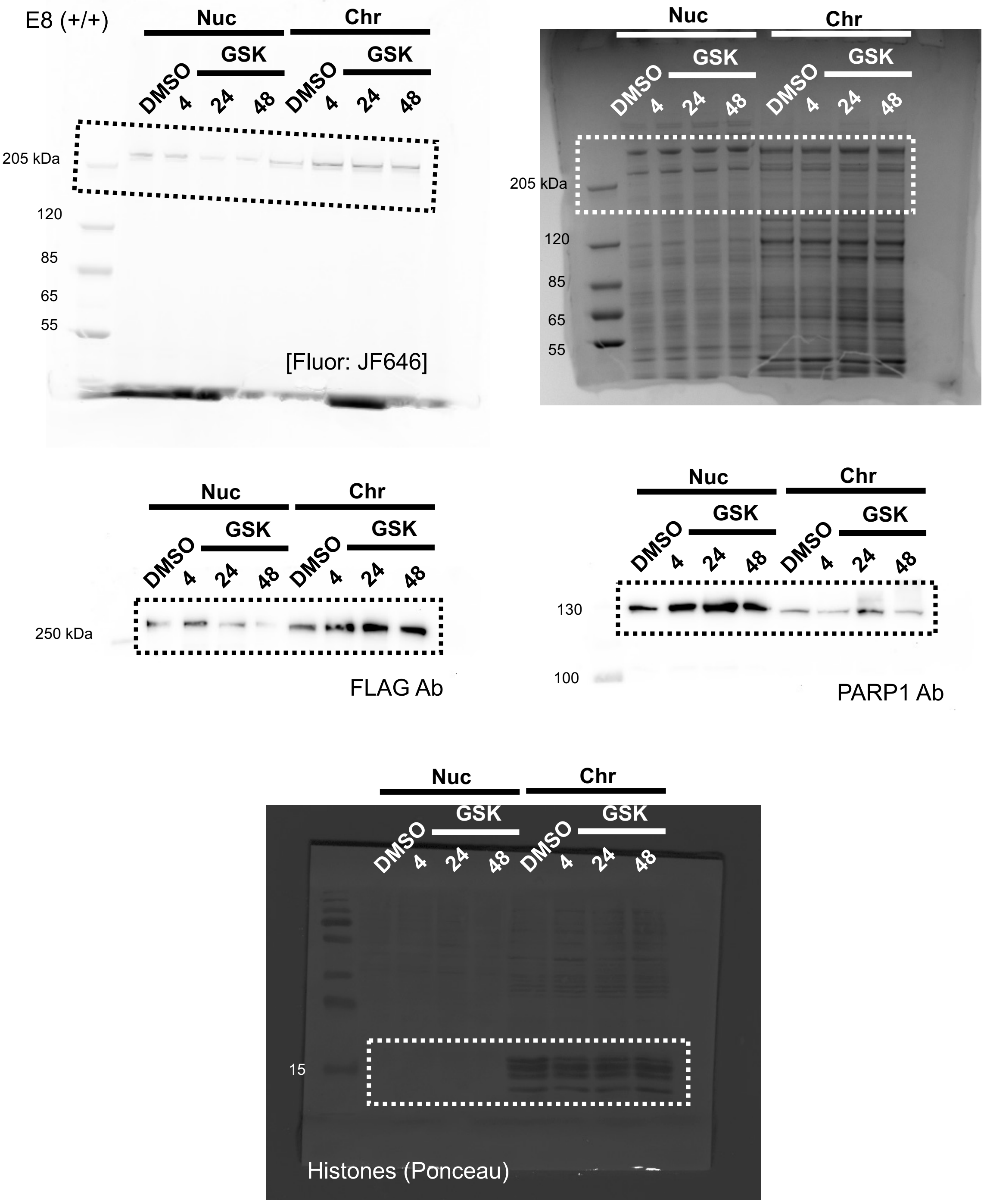

Lee_FigS12

Supplementary Fig S7C

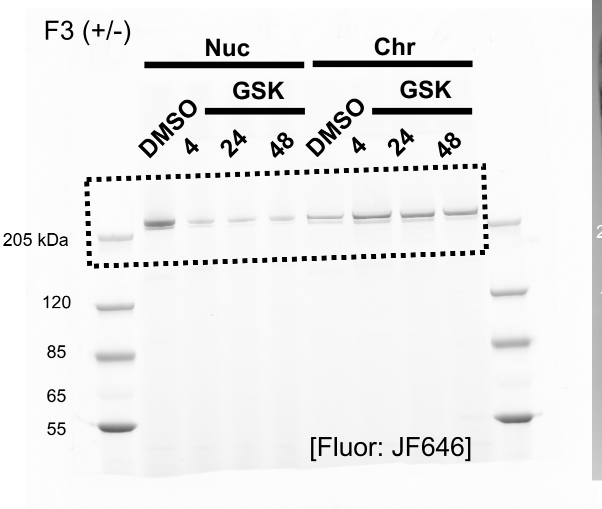

Coomassie

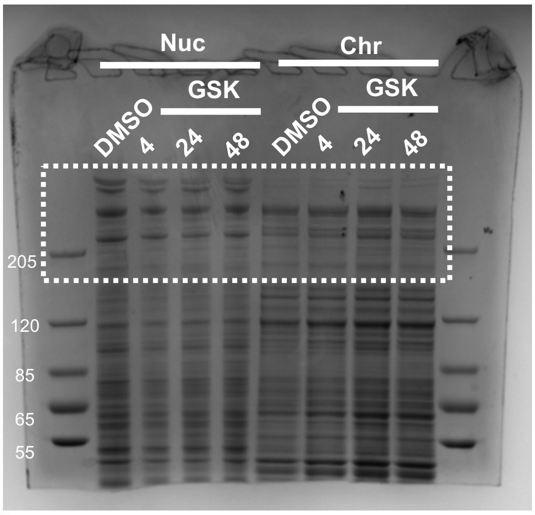

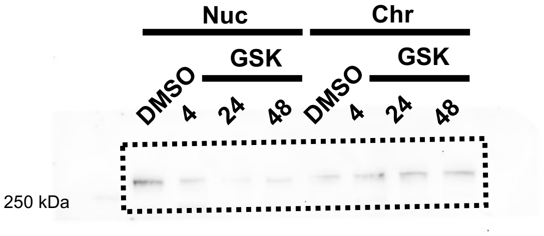

FLAG Ab

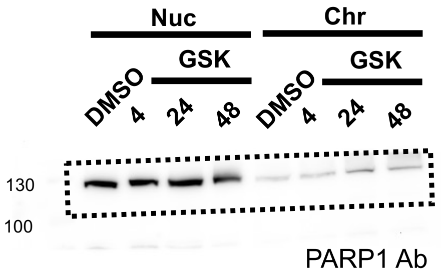

PARP1 Ab

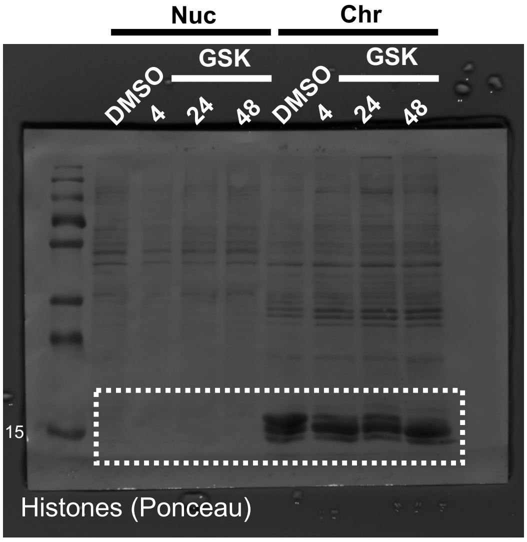

Lee_FigS12

Fig 6B

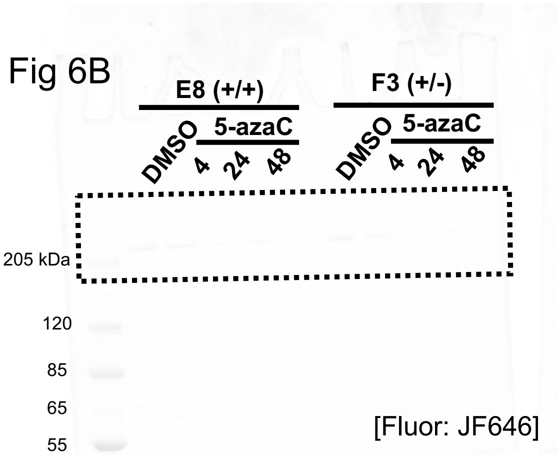

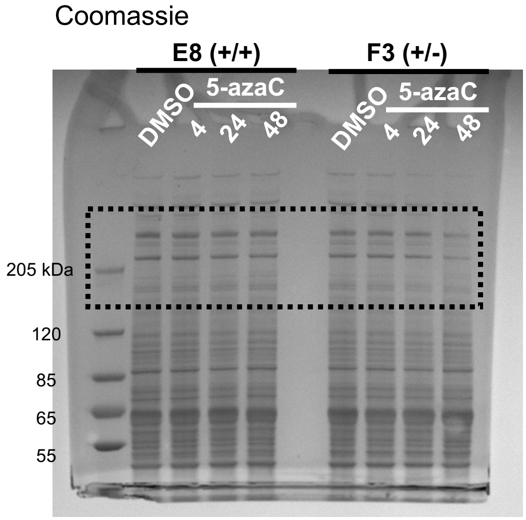

Supplementary Fig S7B

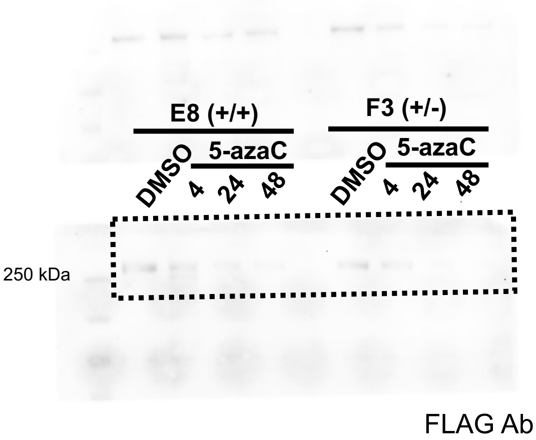

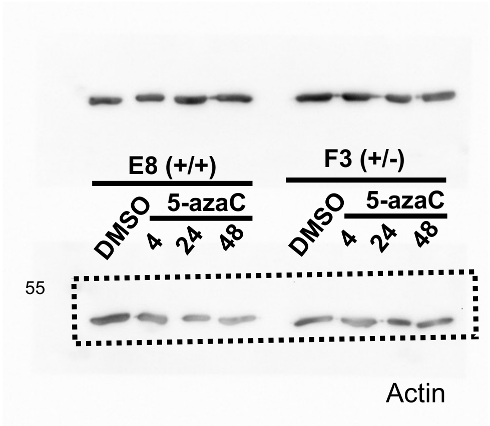

Supplementary Fig S7C

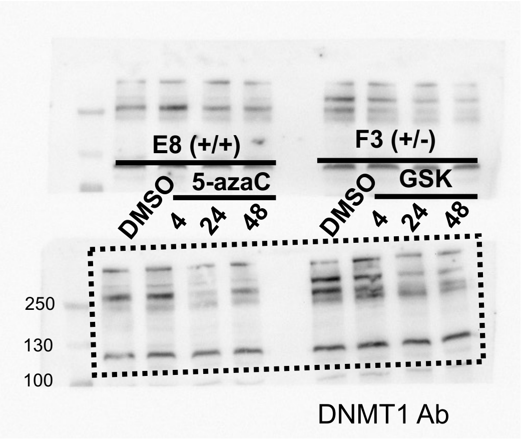

Lee_FigS12

Fig 6G

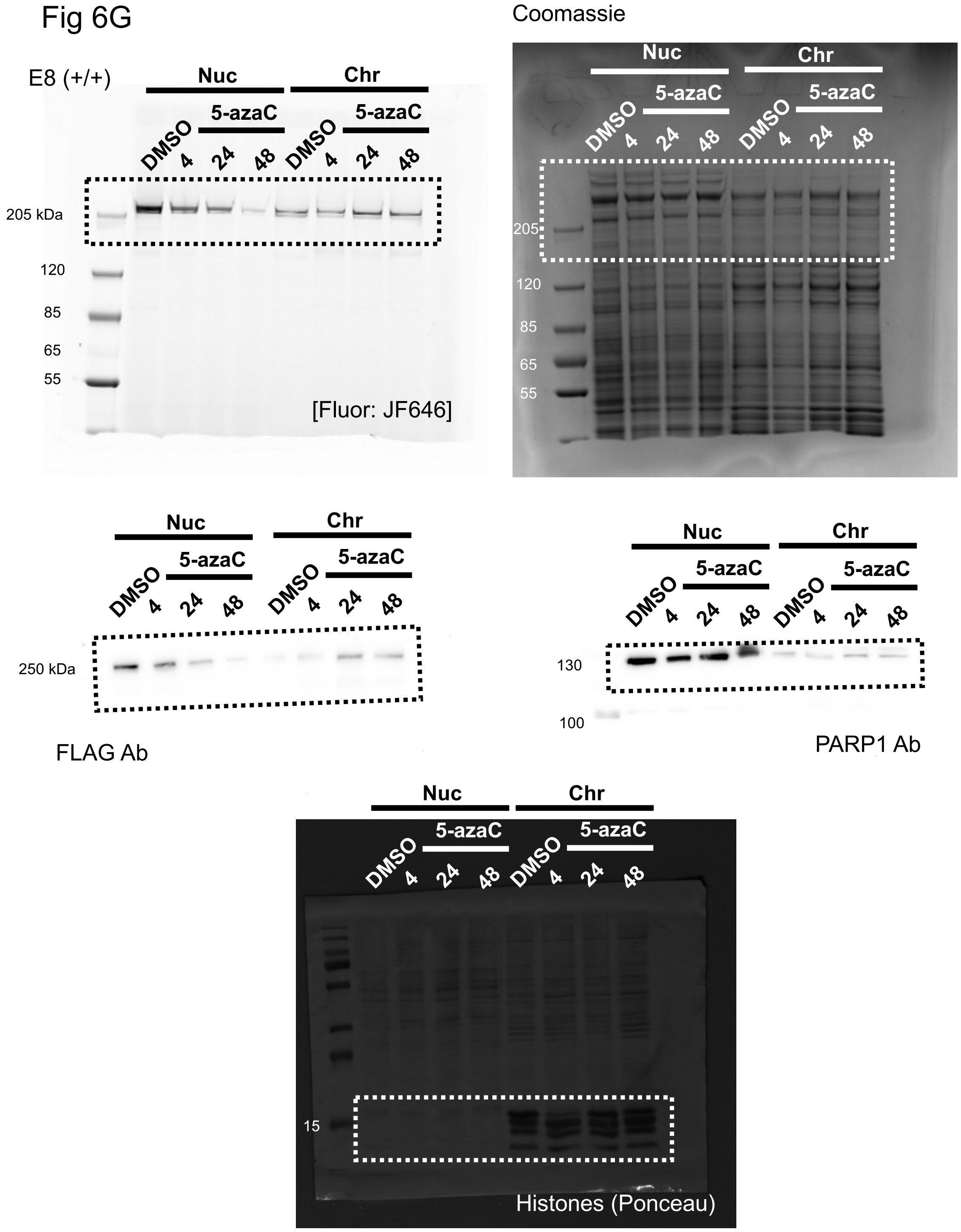

Supplementary Fig S8C

Lee_FigS12

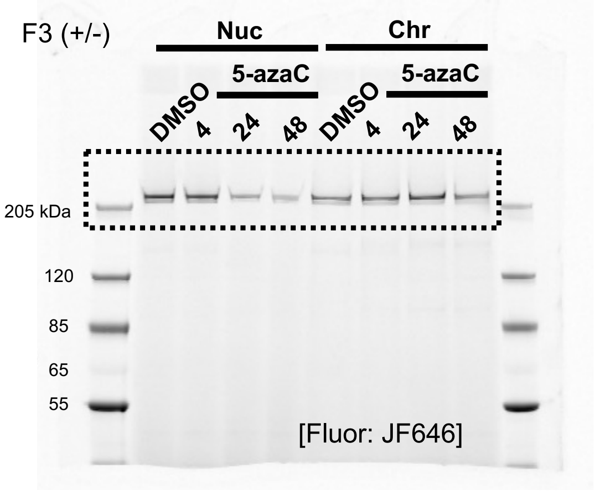

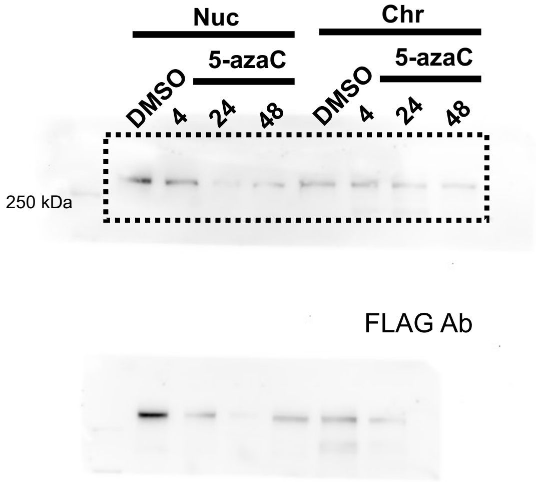

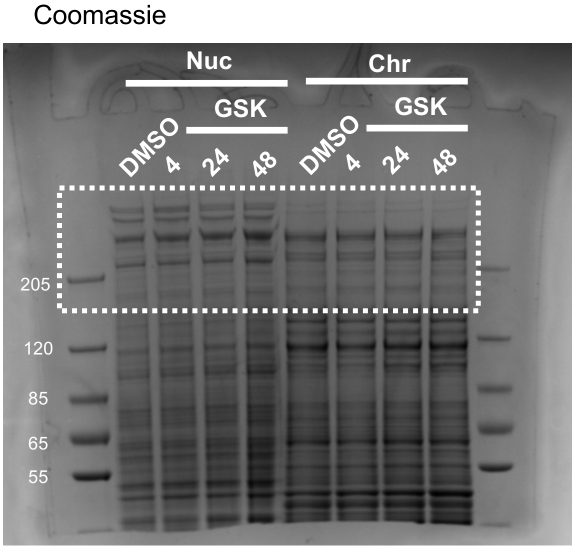

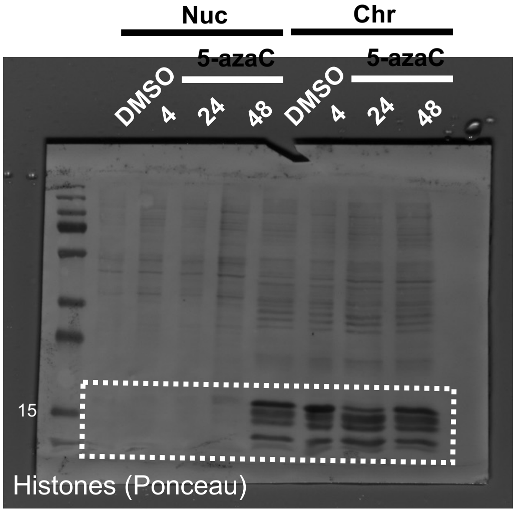

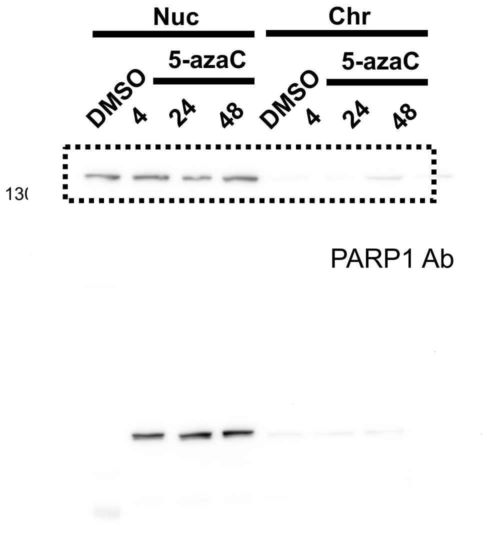

Supplementary Methods

CRISPR editing confirmation PCR

PCR was used to confirm successful CRISPR editing. Firstly, cells were trypsinized and genomic DNA was extracted as in (1). In brief, cells were lysed and digested overnight at 37 °C in a buffer containing 0.2% SDS and 100 µg/mL proteinase K. DNA was then precipitated in 100% isopropanol. For PCR reactions, 50 ng genomic DNA was incubated with 1x Phusion HF buffer, 200 µM each dNTP, 500 nM forward and reverse primers, and 0.2 U/µL Phusion High-Fidelity DNA Polymerase (Thermo Fisher Scientific, #F530L) in an Applied Biosystems 2720 Thermal Cycler.

Primer set sequences were as follows: 1F 5'- CCAAATCTGACTGACACACTTTG-3', 1R 5'- GTCCATTCACTTCCCGGTTG-3', 2F 5'- TATGGAAGGCTCGAGTGGGA-3', 2R 5'- AAACAAGTTGCTAGCTGGTTTATAG-3', 3R 5'-GGTCCAAGTTTAACTGCCCC-3'.

Nanopore sequencing and analysis of DNA methylation

Nanopore sequencing genome skimming (2) was used to monitor DNMT1 inhibition in cells. Following drug treatment, cells were trypsinized and genomic DNA was extracted as described above. Genomic DNA was quantified using Thermo Fisher Qubit 1xdsDNA High Sensitivity Assay Kit per manufacturer's instructions. DNA quality was measured by A260/280 and A260/230 ratios using a Thermo Scientific NanoDrop ONEc. DNA size distribution was measured using Agilent Bioanalyzer 2100 DNA 12000 chip per manufacturer's instructions. 400 ng genomic DNA was carried forward to library preparation. Libraries were prepared using Oxford Nanopore Technologies Native Barcoding Kit 24 V14 (SQK-NBD114.24) per manufacturer's instructions. Libraries were barcoded, pooled, and loaded onto an Oxford Nanopore Technologies MinION Flow Cell (R10.4.1, FLO-MIN114). Libraries were sequenced for 48 h at a rate of 400-450 bases per second with live basecalling off. Approximately 500 Mb of data per genome were collected, representing ~0.1x genome coverage, allowing for an accurate and precise measure of global DNA methylation (2). For post-sequencing analysis, data were basecalled on the command-line interface of the University of Colorado Boulder BioFrontiers Institute FIJI Compute Cluster. Basecalling was performed using Dorado v0.7.4. Reads were aligned to the GRCh38 human genome assembly using Samtools v1.3.1. Methylation data were extracted using the Dorado v0.7.4 modkit pileup command. Downstream analyses were conducted in R version 2024.12.0+467.

Cloning of DNMT1 N-terminal mutants

3xFLAG-DNMT1-Halo was cloned into the pcDNA3 mammalian vector. To generate DNMT1 N-terminal mutants, we custom ordered DNA fragments where all Asp (D) and Glu (E) residues were mutated into Ala (A) (Twist Biosciences, USA) within the indicated region. The vector backbone was amplified using PCR from the 3xFLAG-DNMT1-Halo pcDNA3 plasmid using Phusion DNA polymerase (Thermo Fisher Scientific, USA) according to manufacturer's instructions. For D/E-22-400 and D/E-100-400 mutants, the primer pairs Twist-PS-F' 5'- CATGGTACCCTTGTCATCGTCAT -3', Twist-PS-R' CAGCACAAACTGACCTGCTTCA - 3' were used. For the D/E-22-91 mutant, the primer pairs Twist-PS-F' and DMAP-Twist-R' 5'-TTGGAGAACGGTGCTCATGC -3' were used. The PCR products were separated on a 1X TBE 1% gel and gel purified using E.Z.N.A. Cycle Pure Kit (#D6492-02, Omega Biotek, USA) according to manufacturer's instructions. NEBuilder HiFi DNA Assembly (#E5520S, NEB, USA) was used to ligate the Twist Bioscience DNA fragments and amplified vector backbone according to manufacturer's instructions. Subsequently, the ligated product was transformed into NEB 5-alpha Competent E. *coli* cells (NEB, #C2987) and screened for positive hits by Sanger sequencing.

Flow cytometry of Halo-tagged protein to estimate cellular abundance

To estimate the cellular abundance of our Halo-tagged DNMT1, we used the flow cytometry approach described in (3,4). The Halo-CTCF tagged U2OS cell line (generous gift from Tijan and Darzacq labs, UC Berkeley, USA) was used a standard; it has ~109800 Halo-CTCF molecules per cell. Briefly, 60 mm dishes of Halo-tagged cell lines were incubated with or without 500 μM Halo JF646 ligand for 30 min, the cells harvested and filtered. The samples were passed through the Accuri flow cytometer (BD Biosciences) using the APC-A filter (ex 650 nm) and 10,000 events were recorded. For each biological replicate, two different mean fluorescence intensity measurements were obtained. Subsequently, all data were processed and analyzed using FlowJo v10 Flow cytometry software.

To determine the number of Halo-tagged DNMT1 molecules in each cell line, the mean fluorescence intensity (MFI) of unlabeled cells was first subtracted from that of the labeled cells (Supplementary Table 1). Next, the baseline MFI of the U2OS parental line cell was subtracted from that of the DNMT1-Halo cell lines. Given that Halo-CTCF tagged cells have ~109800 molecules per cell and given their MFI reading from flow cytometry, the number of Halo-tagged DNMT1 molecules was calculated (Supplementary Table 2). From

immunoblotting of DNMT1 (Fig 1C), the levels of endogenous and Halo-tagged DNMT1 were quantified using Gel Analyzer v19.1 and normalized to total proteins visualized by Ponceau staining (which served as a loading control). The number of DNMT1 molecules in the U2OS parental cell line was compared to E8 (+/+) or F3 (+/-) using the Western blot. Next, using flow cytometry estimates of the number of Halo-tagged DNMT1 molecules in E8 (+/+) or F3 (+/-) cell lines, we calculated the number of endogenous DNMT1 molecules in the U2OS parental and F3 (+/-) cell lines (Supplementary Table 3).

Synchronization of cells to G2/M phase

To synchronize cells to G2/M, a protocol modified from (5) was performed. Cells were treated with 20 mM thymidine for 24 h, washed and allowed to recover for 7-9 h, and then 9 μM RO-3306 (Selleck Chemicals, USA, #S7747) was added for 15-18 h. Cells were released from the block by washing with PBS. To determine if the G2/M synchronization worked, the number of microtubule organizing centers (MTOCs) was determined as follows. Cells were plated onto coverslips and synchronized to G2/M. Coverslips were fixed using a 1:1 mixture of ice-cold methanol and acetone for 10 min in -20 °C. The cells were recovered and rehydrated with three separate PBS washes, each time incubating for 5 min, and then incubated overnight with 1:5000 γ-tubulin GTU-88 antibody (Sigma-Aldrich, #T6557) in PBST (1x PBS with 0.1% Triton) with 1 mM BSA. The next day, coverslips were washed 3 times with PBST, each time incubating for 5 min. 1:5000 Mouse Alexa-488 secondary antibody (ThermoFisher Scientific, #A-31571) was incubated for 2 h at room temperature in PBST with 1 mM BSA. Coverslips were washed 3X with PBS-T and mounted as described above. The cells were imaged and the number of MTOCs was counted and scored.

Supplementary Table 1. Mean fluorescence intensity for flow cytometry experiments.

Rep 1	**Labelled MFI***		**Unlabelled MFI***		**MFI* (labelled - unlabelled)**		**Subtract K27 background**	
	**Rep1.1**	**Rep1.2**	**Rep1.1**	**Rep1.2**	**Rep1.1**	**Rep1.2**	**Rep1.1**	**Rep1.2**
**U2OS**	1567	1413	570	575	997	838	n/a	n/a
**CTCF-Halo**	42368	41535	520	525	41848	41010	40851	40172
**E8 (+/+)**	59194	54482	578	579	58616	53903	57619	53065
**F3 (+/-)**	33248	32379	600	588	32648	31791	31651	30953

Rep 2	**Labelled MFI***		**Unlabelled MFI***		**MFI* (labelled - unlabelled)**		**Subtract K27 background**	
	**Rep2.1**	**Rep2.2**	**Rep2.1**	**Rep2.2**	**Rep2.1**	**Rep2.2**	**Rep2.1**	**Rep2.2**
**U2OS**	5321	4180	669	610	4652	3570	n/a	n/a
**CTCF-Halo**	47055	45193	560	634	46495	44559	41843	40989
**E8 (+/+)**	73347	66568	612	961	72735	65607	68083	62037
**F3 (+/-)**	42883	40641	603	646	42280	39995	37628	36425

*MFI = mean fluorescence intensity
Flow cyto # of molecules for CTCF/109800 = Flow cyto # of molecules sample/x molecules
# of molecules of CTCF-Halo in U2OS cells = 109800

Supplementary Table 2. Number of Halo-tagged molecules per cell**.**

		**# of molecules per cell**				
	**Rep1.1**	**Rep1.2**	**Mean Rep1**	**Rep2.1**	**Rep2.2**	**Mean Rep2**
**CTCF-Halo**	**109800**					
**E8 (+/+)**	154869	145040	149955	178656	166183	172420
**F3 (+/-)**	85072	84602	84837	98739	97574	98157

Supplementary Table 3. Estimated # of endogenous DNMT1 (from Western blot & Flow cytometry).

	**# Halo of molecules (Corrected using Western blot)**		**Estimated # of endogenous DNMT1**
	Rep1	Rep2	
**U2OS**			274515
**E8 (+/+)**	149955	172420	
**F3 (+/-)**	84837	98157	81249
**CTCF-Halo**	**109800**		

Supplementary Table 4. Diffusion coefficients of freely diffusing proteins determined by single-particle tracking, comparing DNMT1 to a sample of previously studied nuclear proteins (Related to Fig 2G).

**Protein**	**Calculated MW with Halo (kDa)[a]**	**Cell type**	**D ($\mu m^2/s$)**	**Inverse cubic root MW[b]**	**Reference**
NBS1-Halo	118	U2OS	1.8	0.204	(9)
Halo-MDC1	260	U2OS	1	0.157	(9)
RNF168-Halo	98	U2OS	1.9	0.217	(9)
RNF169-Halo	110	U2OS	2.9	0.209	(9)
Halo-53BP1	247	U2OS	1.1	0.159	(9)
RIF1-Halo	307	U2OS	1	0.148	(9)
Halo-SHLD3	62	U2OS	2.2	0.253	(9)
REV7-Halo	57	U2OS	1.4	0.259	(9)
Halo-SHLD2	127	U2OS	1.6	0.199	(9)
Halo-HLD1	56	U2OS	3.6	0.261	(9)
Halo-DNA-PKcs	502	U2OS	1.7	0.126	(9)
Halo-TRF1	83	U2OS, HeLa	1.5, 1.5	0.229	(10)
Halo-TRF2	93	U2OS, HeLa	1.8, 1.8	0.221	(10)
Halo-TIN2	83	U2OS, HeLa	1.7, 1.8	0.229	(10)
Halo-TPP1	82	U2OS	1.6	0.230	(11)
Halo-POT1	104	U2OS, HeLa	1.6, 1.6	0.212	(10)
DNMT1-Halo	216	U2OS	2.3	0.167	This work
Halo-EZH2	118	U2OS	2.1	0.204	(12)
Halo-SUZ12	116	U2OS	2	0.205	(12)
Halo-RTEL1	167	HeLa	1.5	0.182	(3)
Halo-PARP1	146	U2OS	2.9	0.190	(13)
Halo-PARP2	99	U2OS	3.3	0.216	(13)
Halo-SOX2	67	mESC	2.7	0.246	(14)
HaloTag-3xNLS	34	U2OS	3.9	0.309	(9)
Rpb1-Halo (Pol II)	547	Yeast	0.96	0.122	(15)
Halo-TBP	61	Yeast	2.4	0.254	(15)
Taf1-Halo (TFIID)	1234	Yeast	0.58	0.093	(15)
Halo-Toa1 (TFIIA)	80	Yeast	3.3	0.232	(15)
Sua7-Halo (TFIIB)	72	Yeast	2.6	0.240	(15)
Tfg1-Halo (TFIIF)	190	Yeast	1.5	0.174	(15)
Tfa1-Halo (TFIIE)	128	Yeast	2.1	0.199	(15)

Ssl2-Halo (TFIIH)	567	Yeast	1.2	0.121	(15)
Med14-Halo (MED)	1034	Yeast	0.57	0.099	(15)

[a] Mass includes the protein of interest plus the HaloTag (33 kDa)

[b] Inverse cubic root molecular weight = $\frac{1}{\sqrt[3]{MW}}$

References (for Supplementary methods and tables)

1. Laird PW, Zijderveld A, Linders K, Rudnicki MA, Jaenisch R, Berns A. Simplified mammalian DNA isolation procedure. Nucleic Acids Res. 1991;19(15):4293.
2. Faulk C. Genome skimming with nanopore sequencing precisely determines global and transposon DNA methylation in vertebrates. Genome Res. 2023 June;33(6):948–56.
3. Wu G, Taylor E, Youmans DT, Arnoult N, Cech TR. Rapid dynamics allow the low-abundance RTEL1 helicase to promote telomere replication. Nucleic Acids Res. 2025 Mar;53(5).
4. Cattoglio C, Pustova I, Walther N, Ho JJ, Hantsche-Grininger M, Inouye CJ, et al. Determining cellular CTCF and cohesin abundances to constrain 3D genome models. eLife. 2019 June;8.
5. Renshaw MJ, Panagiotou TC, Lavoie BD, Wilde A. CDK11p58–cyclin L1β regulates abscission site assembly. J Biol Chem. 2019 Dec;294(49):18639–49.
6. Pradhan S, Bacolla A, Wells RD, Roberts RJ. Recombinant Human DNA (Cytosine-5) Methyltransferase. J Biol Chem. 1999 Nov;274(46):33002–10.
7. Bacolla A, Pradhan S, Roberts RJ, Wells RD. Recombinant Human DNA (Cytosine-5) Methyltransferase. J Biol Chem. 1999 Nov;274(46):33011–9.
8. Jansson-Fritzberg LI, Sousa CI, Smallegan MJ, Song JJ, Gooding AR, Kasinath V, et al. DNMT1 inhibition by pUG-fold quadruplex RNA. 2023; Available from: http://www.rnajournal.org/cgi/doi/10.1261/rna
9. Heyza JR, Mikhova M, Bahl A, Broadbent DG, Schmidt JC. Systematic analysis of the molecular and biophysical properties of key DNA damage response factors. eLife. 2023 June;12.
10. Janovič T, Perez GI, Schmidt JC. TRF1 and TRF2 form distinct shelterin subcomplexes at telomeres [Internet]. bioRxiv; 2024. Available from: http://biorxiv.org/lookup/doi/10.1101/2024.12.23.630076
11. Schmidt JC, Zaug AJ, Cech TR. Live Cell Imaging Reveals the Dynamics of Telomerase Recruitment to Telomeres. Cell. 2016 Aug;166(5):1188-1197.e9.
12. Youmans DT, Schmidt JC, Cech TR. Live-cell imaging reveals the dynamics of PRC2 and recruitment to chromatin by SUZ12-associated subunits. Genes Dev. 2018 June;32(11–12):794–805.
13. Mahadevan J, Jha A, Rudolph J, Bowerman S, Narducci D, Hansen AS, et al. Dynamics of endogenous PARP1 and PARP2 during DNA damage revealed by live-cell single-molecule imaging. iScience. 2023 Jan;26(1).
14. Liu Z, Legant WR, Chen BC, Li L, Grimm JB, Lavis LD, et al. 3D imaging of Sox2 enhancer clusters in embryonic stem cells. eLife. 2014;3:e04236.

15. Nguyen VQ, Ranjan A, Liu S, Tang X, Ling YH, Wisniewski J, et al. Spatiotemporal coordination of transcription preinitiation complex assembly in live cells. Mol Cell. 2021 Sept;81(17):3560-3575.e6.

Supplementary movies files

All movie files shown are ~ 97 fps and 10 s in duration and taken with two Andor Ixon 897 EMCCD cameras, scale bar = 5 μm.

SM1) Halo-tagged DNMT1 molecules in asynchronous E8 (+/+) cells, labelled with JF646 Halo ligand.

SM2) Halo-tagged DNMT1 molecules in asynchronous F3 (+/-) cells, labelled with JF646 Halo ligand.

SM3) Halo-tagged DNMT1 molecules in E8 (+/+) cells 1 h post thymidine release (early S phase), labelled with JF646 Halo ligand.

SM4) Halo-tagged DNMT1 molecules 5 h post thymidine release (mid S phase) in E8 (+/+) cells, labelled with JF646 Halo ligand.

SM5) Halo-tagged DNMT1 molecules in asynchronous E8 (+/+) cells, labelled with JF646 Halo ligand.

SM6) Halo-tagged DNMT1 molecules 1 h post RO-3306 release (G2/M phase) in E8 (+/+) cells, labelled with JF646 Halo ligand.

SM7) Halo-tagged DNMT1 molecules in wildtype DNMT1 (transfected) in U2OS cells, labelled with JF646 Halo ligand.

SM8) Halo-tagged DNMT1 molecules in D/E-22-400 DNMT1 mutant (transfected) in U2OS cells, labelled with JF646 Halo ligand.

SM9) Halo-tagged DNMT1 molecules in D/E-100-400 DNMT1 mutant (transfected) in U2OS cells, labelled with JF646 Halo ligand.

SM10) Halo-tagged DNMT1 molecules in D/E-22-91 DNMT1 mutant (transfected) in U2OS cells, labelled with JF646 Halo ligand.

SM11) Halo-tagged DNMT1 molecules in DMSO treated E8 (+/+) cells, labelled with JF646 Halo ligand.

SM12) Halo-tagged DNMT1 molecules 4 h following GSK treatment in E8 (+/+) cells, labelled with JF646 Halo ligand.

SM13) Halo-tagged DNMT1 molecules in DMSO treated E8 (+/+) cells, labelled with JF646 Halo ligand.

SM14) Halo-tagged DNMT1 molecules 4 h following 5-azaC treatment in E8 (+/+) cells, labelled with JF646 Halo ligand. An example of a cell with 'slow moving' DNMT1 molecules is shown.

SM15) Halo-tagged DNMT1 molecules in DMSO treated E8 (+/+) cells, labelled with JF657 Halo ligand.

SM16) Halo-tagged DNMT1 molecules 4 h following decitabine treatment in synchronized E8 (+/+) cells, labelled with JF657 Halo ligand.
